# Confronting complexity and supporting transformation through health systems mapping: a case study

**DOI:** 10.1186/s12913-021-07168-8

**Published:** 2021-10-23

**Authors:** Anna J. Hussey, Shannon L. Sibbald, Madonna Ferrone, Alyson Hergott, Robert McKelvie, Cathy Faulds, Zofe Roberts, Andrew D. Scarffe, Matthew J. Meyer, Susan Vollbrecht, Christopher Licskai

**Affiliations:** 1 Asthma Research Group Windsor-Essex County Inc., Windsor, ON Canada; 2 Faculty of Health Sciences, Western University, London, ON Canada; 3 Department of Family Medicine, Schulich School of Medicine and Dentistry, Western University, London, ON Canada; 4 Hotel-Dieu Grace Healthcare, Windsor, ON Canada; 5 St. Joseph’s Health Care, London, ON Canada; 6 Cardiology, Schulich School of Medicine and Dentistry, Western University, London, ON Canada; 7 Telfer School of Management, University of Ottawa, Ottawa, ON Canada; 8 London Health Sciences Centre, London, ON Canada; 9 Department of Epidemiology and Biostatistics and Interfaculty Program in Public Health, Schulich School of Medicine and Dentistry, Western University, London, ON Canada; 10 Ivey Business School, London, ON Canada; 11 Lawson Health Research Institute, London, ON Canada; 12 Respirology, Schulich School of Medicine and Dentistry, Western University, London, ON Canada

**Keywords:** Health care reform, Ecosystem maps, Co-creation, Chronic obstructive pulmonary disease, Heart failure, Health system navigation, Integrated delivery system

## Abstract

**Introduction:**

Health systems are a complex web of interacting and interconnected parts; introducing an intervention, or the allocation of resources, in one sector can have effects across other sectors and impact the entire system. A prerequisite for effective health system reorganisation or transformation is a broad and common understanding of the current system amongst stakeholders and innovators. Chronic obstructive pulmonary disease (COPD) and heart failure (HF) are common chronic diseases with high health care costs that require an integrated health system to effectively treat.

**Study description:**

This case study documents the first phase of system transformation at a regional level in Ontario, Canada. In this first phase, visual representations of the health system in its current state were developed using a collaborative co-creation approach, and a focus on COPD and HF. Multiple methods were used including focus groups, open-ended questionnaires, and document review, to develop a series of graphical and visual representations; a health care ecosystem map.

**Results:**

The ecosystem map identified key sectoral components, inter-component interactions, and care requirements for patients with COPD and HF and inventoried current programs and services available to deliver this care. Main findings identified that independent system-wide navigation for this vulnerable patient group is limited, primary care is central to the accessibility of nearly half of the identified care elements, and resources are not equitably distributed. The health care ecosystem mapping helped to identify care gaps and illustrates the need to resource the primary care provider and the patient with system navigation resources and interdisciplinary team care.

**Conclusion:**

The co-created health care ecosystem map brought a collective understanding of the health care system as it applies to COPD and HF. The map provides a blueprint that can be adapted to other disease states and health systems. Future transformation will build on this foundational work, continuing the robust interdisciplinary co-creation strategies, exploring predictive health system modelling and identifying areas for integration.

**Supplementary Information:**

The online version contains supplementary material available at 10.1186/s12913-021-07168-8.

## Introduction

### Overview

The progression of modern medicine has escalated the complexity of health care systems [1] and with it exacerbated the challenge of achieving integration and optimal performance of health system components. Canada has a universal public health care system; Ontario, Canada’s most populous province with 14.8 million residents [2], is introducing a new model of regional health teams to enhance the integration of care delivery across sectors and facilitate cohesive functioning of the system as a whole [3]. The overarching provincial goal of these teams (called Ontario Health Teams, or OHTs) is to build a modern, sustainable, integrated health care system that provides coordinated interdisciplinary care through shared leadership, resources, tools, and performance expectations [4]. The expected health system impacts are those defined by the quadruple aims of: improving patient and caregiver experience; improving the health of populations; reducing per capita cost; and improving the work life of providers [5, 6]. The transformation emphasizes upstream care in the community which has been demonstrated to improve patient outcomes and health system performance [7–12].

The province-wide health system transformation is substantial with the formation of up to 100 OHTs. In the Southwestern region of the province, health care providers in the London-Middlesex region are collaborating to develop and implement the Middlesex London Ontario Health Team (MLOHT). The MLOHT has committed accountability for the primary and secondary health needs of a fully attributed population of over 514,000 people. The current focus of MLOHT is on system integration that supports patients with chronic obstructive pulmonary disease (COPD) and heart failure (HF) who are at risk of avoidable hospitalization and most specifically complex patients who require coordinated system-level care solutions.

### Disease focus

The global burden of chronic disease is substantial; chronic diseases dominate the ten leading causes of death [13], and these trends are locally confirmed in Ontario chronic disease data [14]. COPD and HF, two high burden chronic diseases, had a respective prevalence among Ontario’s adult population of 904,940 (10.3%) and 278,530 (3.7%) in 2016 [15]. Patients presenting with these diseases are predominantly managed in primary care and are usually older with multiple health impacts with which to contend [9, 10]. In addition, COPD and HF are associated with high acute health service use; in 2019 there was a combined total of over 56,000 hospitalizations, 5% of all hospitalizations in Ontario and 9.7% of hospitalizations for individuals aged 65 years and older [16]. These two disease states have been identified as a population in need of system-level care coordination and supported primary care management [9, 10].

### Complex system methodology

There is no doubt that health care systems demonstrate complex system characteristics, such as a multitude of interactions, unpredictable and non-linear responses to these interactions, an ability to intrinsically adapt or self-organise and the notion that the sum of individual interactions does not equate to the overall properties exhibited by the system [17–23]. They are open systems with permeable boundaries, that do not reach equillibrium and are nested, with complex units embedded within the complex system [17, 21–23].

There is a large body of literature describing frameworks and approaches to evaluate complex systems and facilitate system level change [24–29]. A recent review examined public health evaluations exploring the different complex system methodologies used and the utility of evidence generated [27]. Of 75 studies reviewed, 45 evaluations reported some form of system mapping and these diagrammatic representations were frequently produced collaboratively by stakeholders. Most of these studies used the generated system map as a tool to provide a framework for further modelling [27].

One systems approach applied to health care is collaborative modelling, defined as, “the joint creation of a systems representation with the participation of end users, stakeholders, experts and analysts” ([28]p. 16). The reported role of a collaborative model is to illustrate the real-world functioning of a system, to explore proposed implementation impact through simulation and to help stakeholders develop a common understanding of the system, the causes of problems that exist, and the availability of mechanisms to promote change [27–29]. A unified first stage of these methodologies is to examine the current system and the problems that exist within, often using visual and graphical representations [26–29].

### Study objectives

The objective of this case study was to document and visually represent the current state health care system for individuals with COPD and HF with an aim to identify and understand the sectors and their contributions across the biopsychosocial continuum. We sought to foster a common understanding among stakeholders, create shared ownership of the transformation agenda, facilitate a systems approach to change, and in doing so support subsequent phases of the health care system transformation. Despite a principle focus on the practical processes undertaken, in an additional academic objective we endeavour to exemplify how complexity theory underpins both the approach used and the interpretation of the findings.

## Methods

A qualitative case study is a, “study of the particularity and complexity of a single case, coming to understand its activity within important circumstances” ([30] p. 148, 24 p. xi). This case study followed a flexible design based on a central research purpose to assess the current state health care system. As per a Stakian characterization of case study, the case under study is the health care system within London-Middlesex, specifically the MLOHT as it aligns with the definition of a case as a purposeful integrated system with a boundary and working parts [30, 31]. Consistent with the case study methodology, as per Stake (1995), data collection and analysis were conducted simultaneously through direct interpretation (e.g., real-time edits during feedback) and categorical aggregation (e.g., clustering common and emerging themes) [30, 31].

### Case Description

**Fig. 1:**
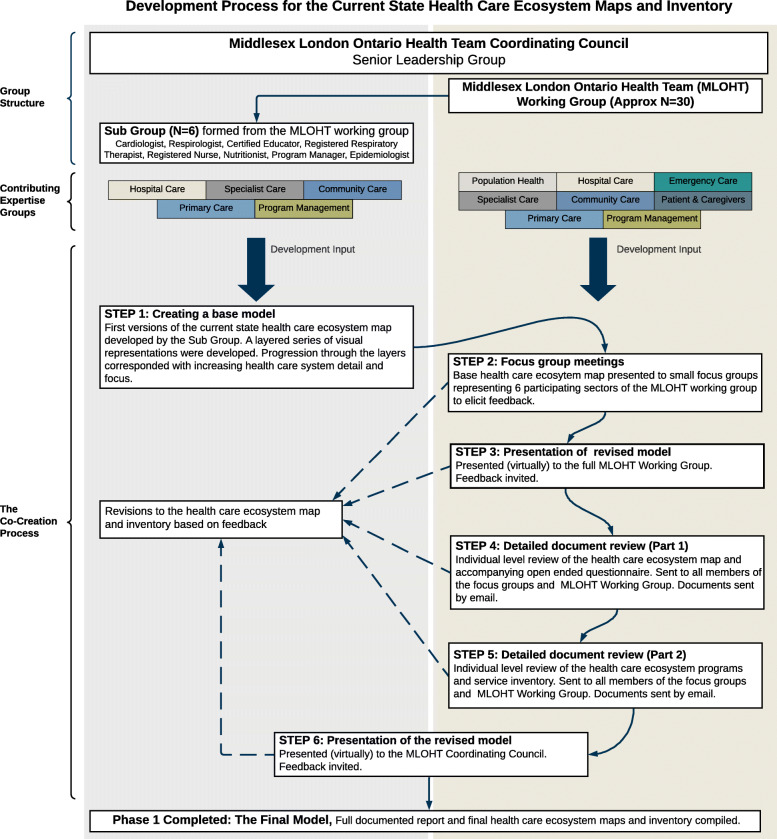
The health team structure, contributing sectors and the co-creation process for phase 1 of health system transformation

(Refer to Fig. 1 for an illustration of the organizational groups described in this section). The MLOHT senior leadership group, the Coordinating Council set up a working group (herein referred to as the Working Group) of over 30 individuals, with a diversity of skills and expertise from all sectors of the health care system, to facilitate and guide the implementation of MLOHT objectives. The Working Group recognized that before implementation of this transformative change an understanding of the current state of the system was needed. The Working Group set out to create a visual representation of the system to identify resource availability, evidence of care gaps, and potential areas of unnecessary system duplication to inform future resource allocation or reallocation. A sub-group of 6 individuals from the Working Group (herein referred to as the Sub-Group) was formed to coordinate this first phase.

We defined sector informally as a segment of the health care delivery system including hospitals, homecare, community and public health, emergency care, long-term care, primary care, specialist care and patient and caregivers. Sector expertise in the Working Group and the Sub-Group included: the hospital, specialist care, community care, primary care and program management. The Working Group also included: population health expertise, patient and caregiver representation and emergency care representation. Patient and caregiver involvement was through the Patient/Client and Care Partner Council (i.e., a group of people outside the health care field, committed to providing a representative voice based on lived experience within the health care system).

A multi-method co-creation approach, combining focus groups, individual open-ended questionnaires, and document review, was used to develop the system visualizations. Co-creation spans a continuum with different levels of user involvement depending on the approach used [32]. Development of the visualizations was coordinated by the Sub-Group, with heavy involvement from the Working Group and limited involvement from the Coordinating Council. The collaborative co-creation steps, outlined below, were spaced at 2–4-week intervals to reduce the burden on contributors (Fig. 1).

#### Step 1 – creating a base model

The members of the Sub-Group co-constructed the first version, or base model, of the current state health system map. Interpretation of more formal modelling approaches such as causal-loop-diagrams, functional resonance analysis or business process models is challenging for a “non-modelling” audience and is further complicated by associated nomenclature [33–35]. Therefore, to achieve broad comprehensibility but without diminishing system complexity, visual representations of the system were developed from a variety of system viewpoints or tiers. The first tier was a “35,000 ft” view and with each of the 4 tiers the focus narrowed giving a more granular depiction of the system. We selected Lucidchart, for it’s accessibility, ease of use, versatility and low-cost. Lucidchart is a web-based platform for diagraming and charting, and was used to present, review, and edit the representations, allowing different layers of complexity to be added to the tiers [36]. The ability to develop the model in tiers created an opportunity for three dimensional modeling of the ecosystem. Collectively these tiers are referred to as the health care ecosystem map (Table 1).

**Table 1:** Health Care Ecosystem Map, Tiers 1–4

**Tier 1:** The main components and interactions within the health care system.	
**Tier 2:** The elements of care	
**Tier 3:** Services and programs available to address the elements of care	
**Tier 4:** Program and service inventory	

#### Step 2 - focus group feedback on major components and interactions

Focus groups were sector-specific and representative individuals (up to 7 per sector) were identified and invited to participate in the collaborative development of the ecosystem health care map. Six sector-specific focus groups were conducted to elicit a first round of feedback on the base model. Most focus group participants were members of the Working Group and/ or Coordinating Council. Each focus group lasted approximately 2-h and started with a 30 to 45-min presentation describing the phase 1 aims and objectives along with the 4 tier base model visual representation. The remainder of the meeting was open-ended discussion on specific feedback for each of the 4 tiers and on the approach as a whole. A member of the Sub-Group took notes to document comments and suggestions. After each focus group a Sub-Group debriefing session was held to discuss ways to address suggested amendments; the health care ecosystem maps were then modified accordingly.

#### Step 3 – presentation of revised model

A virtual presentation and open discussion of the health care ecosystem map was held with the larger Working Group. Feedback from this discussion was added to the health care ecosystem map.

#### Step 4 – detailed document review, tier 1 and 2

The next step was a detailed document review of the revised Tier 1 and Tier 2 maps from Step 2 and 3. A specific accompanying document with fillable open-ended questions was created to elicit detailed responses from the focus group members (Step 2, see additional file 2). Participants in Step 4 were provided an individual copy of the ecosystem map and asked to review the interactions (represented by arrows) in detail and provide feedback. The other questions on the questionnaire were compiled from feedback from Steps 2 and 3, that the Sub-Group was unable to definitively incorporate into the map and instead sought to draw on the breadth of expertise of the Working Group. The health care ecosystem map (Tiers 1 and 2) and accompanying questionnaire were also sent for review to other members of the Working Group who were not involved in the focus groups but attended the virtual presentation (Step 3). Document feedback was due 2–3 weeks after delivery and reminder emails were sent out at 1 week. Feedback from the questionnaires was compiled and revisions were made to the Tier 1 and Tier 2 maps.

#### Step 5 – detailed document review, tier 4

The Tier 4 program and service inventory was distributed (in an excel spreadsheet) to all focus groups invitees and other members of the Working Group for review of accuracy and omissions. Eligibility criteria and any associated fees were included as data fields to document accessibility of the programs and services. The feedback was collected from amendments made to the returned inventory document or comments and suggestions via email. These were combined and the Tier 4 inventory was updated accordingly.

#### Step 6 – compilation and presentation of the final model

The final map was presented to the Coordinating Council with the opportunity to provide feedback. Feedback was built into the map and the final representation images were submitted to the Coordinating Council.

#### Linking complexity theory to practice

Steps 1–6 are a practical description of the health care ecosystem map development process. We then placed this pragmatic case study within the framework of complexity theory to enhance the academic contribution of this work and foster a more fulsome understanding of the attributes of dynamic health ecosystem function.

## Results

### Co-creation contributions

The 6 identified focus groups from Step 2 had representation from primary care, the hospital sector, paramedicine, home and community care, community support services, and patients and caregivers (through the MLOHT Patient/Client and Care Partner Council). Twenty people participated in the focus group (77%) to review the base maps of the health care ecosystem created in Step 1 (Table 2). In total 36 individuals attended presentations of the health care ecosystem, either virtually or in person (Step 2 and Step 3), with the opportunity to provide feedback and contribute to the co-creation process. In Step 4, the Tier 1 and 2 document and open-ended questionnaire was emailed to the 36 individuals; feedback was returned by 12 participants (33% response); feedback was provided from participants from 4 of the 6 focus groups. In Step 5, the Tier 4 inventory was emailed to the same 36 individuals and feedback was returned by 3 (8%, from 1 of the 6 focus groups). The final model was presented to the MLOHT Coordinating Council (Step 6) for an additional opportunity to contribute to the Tier 1 to 4 contents; an additional 22 individuals who had not participated in any of the other groups were also present. The co-creation approach involved over 60 individuals with experience across all sectors of the health system. We estimate the time commitment by the Sub-group (6 people) was 1.5 days per week over a 3 month interval and that other participants contributed between 1 and 4 h of time.

**Table 2:** Contribution to the co-creation approach

	Invited to review (Tier 1–4)	Reviewed (Tier 1–4)	Invited to review (Tier 1&2)	Reviewed (Tier 1&2)	Invited to review (Tier 4)	Reviewed (Tier 4)	Reviewed (Tier 1–4)
	Step 2		Step 3		Step 4		Step 5
Primary Care	5	4^a^	4	2	4	0	–
Hospital Sector	4	3^a^	3	0	3	0	–
Home & Community Care	3	3^a^	3	1	3	0	–
Community Support Services	5	3^a^	3	1	3	3	–
Patient, Client and Care Partner Council	7	7 (3^a^,4^b^)	6	4	6	0	–
Paramedicine	2	0	1	0	1	0	–
MLOHT Working Group	–	16^b^	16	4	16	0	–
MLOHT Coordinating Council	–	–	–	–	–	–	22

MLOHT Middlesex London Ontario Health Team,

V1 version 1, shown in presentation form at the in person focus group meetings or the virtual MLOHT working group meeting.

V2 version 2, sent via email as two documents Tier 1 & 2 with a fillable questionnaire and Tier 4 as an excel spreadsheet.

^a^attended in person

^b^attended virtually

### Examples of actionable contributions

Most of the feedback obtained from the co-creation team was qualitative. Individuals contributed from the perspective of their area of expertise. Edits to the Lucidchart health care ecosystem map were captured in real-time during the focus group meetings or adjusted shortly thereafter. For example, it was suggested by one focus group that the term ‘social services’ in the base model should be removed and replaced with ‘community support services’, a broader umbrella term to capture not only social services but many other relevant community based services available. Additional feedback on this component was gathered in the document review (Step 4) which now returned ‘social services’ and placed it in the primary health care level. Further, using categorical aggregation we identified common themes from the focus group meetings (Step 2) and from the questionnaires (Step 4). As an example, one theme identified in at least three of the five focus groups was the limited accessibility of some of the defined primary care elements. Whereas, the focus group agreed that the care elements as identified existed in the current health care ecosystem map, they identified that access to these care elements was so limited that it was debatable whether to include them in the current state system or not. This is one example where the richness of the contribution to the current state map helped to identify priority evaluation for future planning.

### The health care ecosystem map

**Fig. 2:**
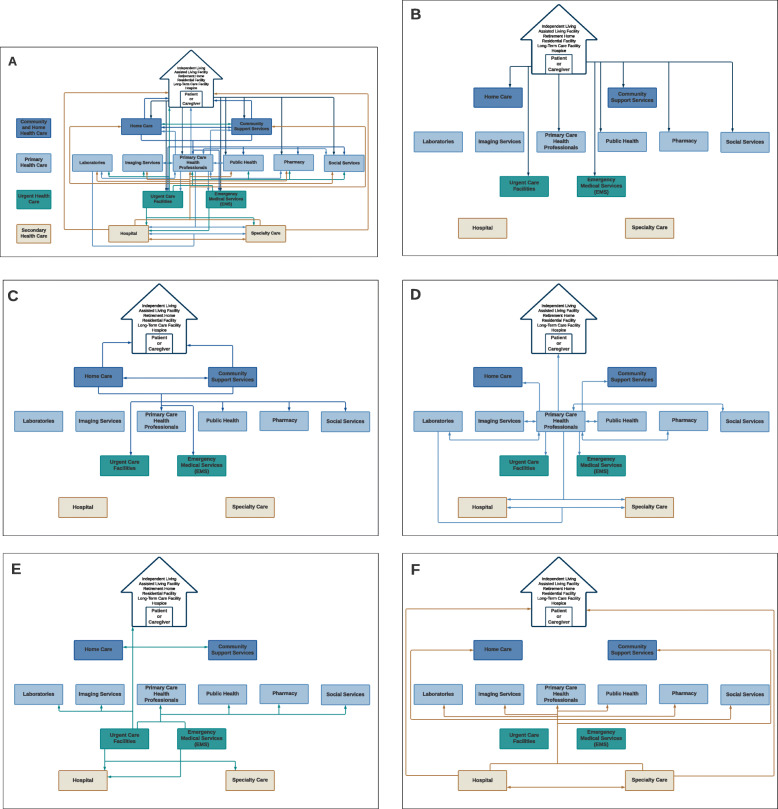
The Final version of the Tier 1 Health care Ecosystem: **A** all interactions visible, **B** interactions originating from the patient or caregiver, **C** from community & home health care, **D** from primary health care, **E** from urgent health care and F from secondary health care

The health care ecosystem map is presented in a static format in Figs. 2 and 4 embedded in the manuscript. Dynamic visual representations of the map is available through the Lucidchart URL link: Dynamic health care ecosystem map

Steps 1 to 6 informed the development of the the health care ecosystem map; the final versions of Tier 1 to 4 are detailed below. Online supplementary files are provided showing the initial version of the health care ecosystem map (additional file 1), questionnaires administered and reviewer responses (additional file 2) and the full regional program and services inventory (additional file 3).

#### Tier 1: the main components and interactions within the health care system (Fig. 2)

Tier 1 identified the main components that make up the generic framework of a health care system, placing the patient and caregiver in their home at the summit of the system, either independent living, an assisted living facility, retirement home, residential facility, nursing home or hospice. The main components identified in Tier 1 were divided into four levels: community and home health care, primary health care, urgent health care, and secondary health care. Arrows, were used to represent interactions, and colours to depict the levels of care from which they originated. An arrow was placed to represent an interaction between one health system component, health care professional or the patient and another health system component, health care professional or the patient. A simple example would be a primary care physician referring a patient to a specialist. The patient comes to the primary care physician and because of that consultation they are referred to a specialist. An arrow is drawn from the patient to the primary care physician and then from the primary care physician to the specialist. There would not be a direct line from the patient to the specialist as there is no direct route for a patient to consult a specialist in this health system.

This tier used different layers to display the interactions originating from the different levels, and these layers could be displayed separately or all together to reduce or increase the complexity as required. Figure 2A-F show the final version of Tier 1. Figure 2A is the most complex form with all layers visible; Figs. 2B-F show individual layers for each care level. There were 61 touchpoints (illustrated by the arrow heads) identified in the most complex version (Fig. 2A), which broke down to 8 for the patient or caregiver, 10 for community and home health care, 19 for primary health care, 12 for urgent health care and 12 for the secondary health care level.

#### Tier 2: the elements of care (Fig. 3)

Tier 2 represents the elements of care a patient with COPD or HF may utilize or require within each component of the system identified in Tier 1. It is acknowledged that while this is presented as the ‘current state’ health care system, not all of the elements of care are accessible to all patients nor are they currently available at all clinics and sites across the region, due to a lack of resources or other health system constraints. The arrows in this tier show the patient pathway through the system with different entry points including routine, urgent or emergent access points.

**Fig. 3:**
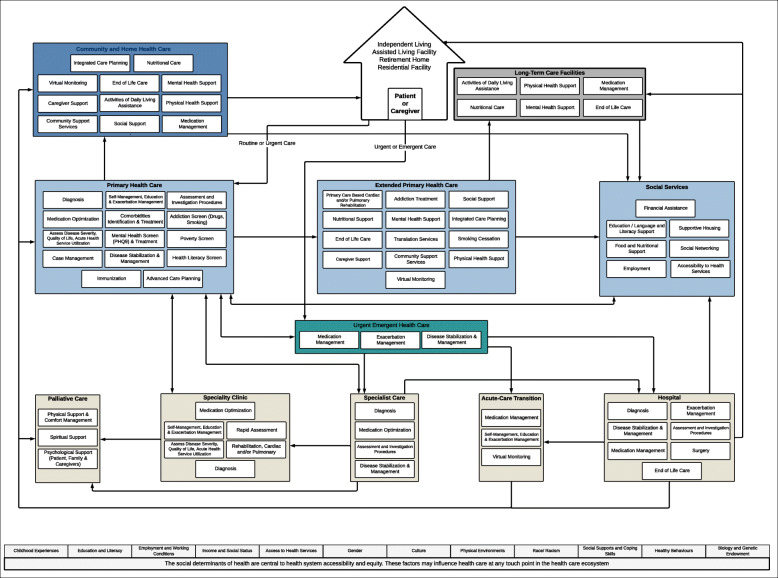
The final version of the Tier 2 Health care Ecosystem showing the elements of care utilised or received by a patient or caregiver in each health system component and possible pathways

The final version of the Tier 2 health care ecosystem is shown in Fig. 3. Tier 3 was modified the most from the base model, it is the most complex layer in terms of visible components and received the most feedback. Long-term care facilities were pulled into a box adjacent to the patient home to recognize their dual role as a home and as a health care provider. The primary health care level was split into three components: primary health care, extended primary health care, and social services. This split distinguished between care delivered in the primary care provider clinic by the key primary care providers (e.g., family physician, nurse practitioner, respiratory therapist, dietician, pharmacist), whereas extended primary health care requires additional resources such as a system navigator or referral to specialist programs and services. After discussion and feedback, the social determinants of health (i.e., as per the Public Health Agency of Canada [37]) were added to the Tier 2 map. The base of the map reflects the importance of considering the elements of health care within these foundational concepts and relates specifically to the COPD and HF population under the social services component of primary health care. During the co-creation process the secondary health care level was further divided into 5 components: hospital, acute-care transition, specialist care, specialty clinic and palliative care. This division gave a distinction between inpatient and outpatient care. There were 11 elements of care identified for home and community care, 6 in long-term care, 34 associated with primary health care, 3 in urgent health care and 23 in the secondary health care level.

#### Tier 3: services and programs to address the elements of care (Fig. 4)

Tier 3 was developed to identify the programs and services regionally available to address the elements of care identified in Tier 2. To simplify this process, services, and programs were reorganized under eight categories of care delivery: pre-diagnosis and primary prevention, diagnostics, maintenance and secondary prevention, exacerbation management, rehabilitation, long-term care, end of life care, and psychosocial support. Regional programs or services were initially identified (in Step 1) using a source document provided by the Working Group and supplemented by publicly available material obtained primarily via web searches. The programs and services were organized under the applicable Tier 2 elements of care.

**Fig. 4:**
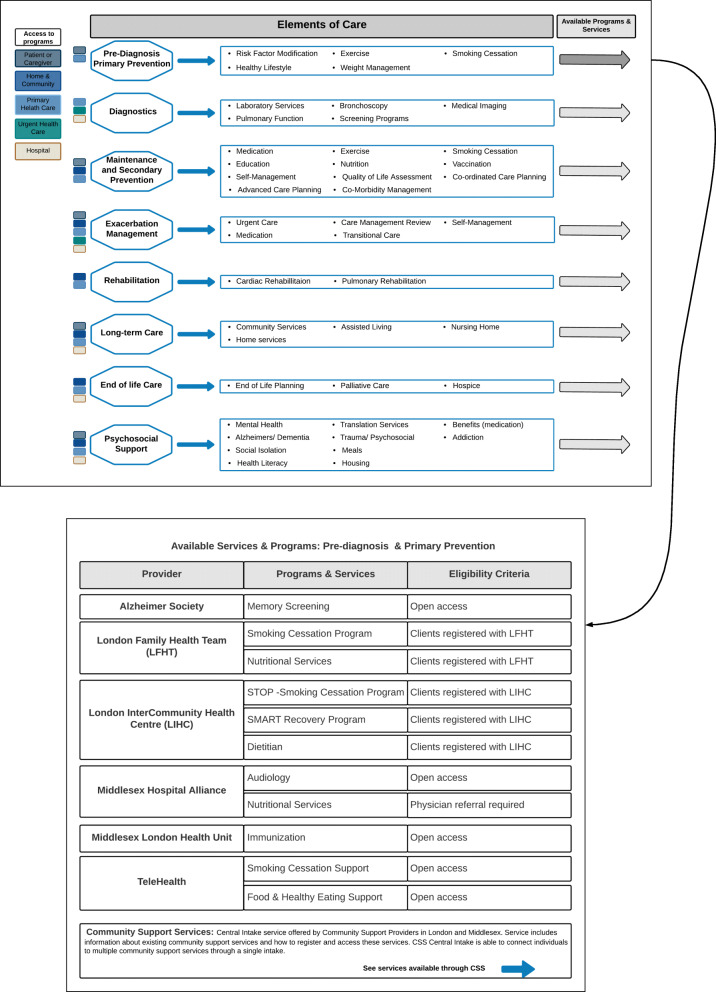
Tier 3 shows the elements of care under broader categories and each category is linked to identified programs and services by interactive buttons (shown for Pre-diagnosis and Primary Prevention)

Tier 3 shows a different viewpoint of the health care ecosystem with content that overlaps both Tier 2 and Tier 4. Tier 3 was adapted into its final version based on direct feedback from Step 2 and 3 and indirect feedback complied from Steps 4 and 5. The view presented in Fig. 4 was developed as an interactive technology-enabled representation displaying the points of access to care. For example, ‘clicking’ on the patient or caregiver would visualize the direct access to some programs and services offered for: pre-diagnosis and primary prevention, maintenance and secondary prevention, exacerbation management, long-term care, and psychosocial support. Clicking the arrow next to each element of care displayed all the related available programs and services. Figure 4 shows an example of this for the pre-diagnosis and primary prevention category.

#### Tier 4: program and service inventory

Tier 4 was a more detailed look at the services and programs identified in Tier 3. An inventory was compiled that broke down the provider, the services and programs offered, the care elements addressed, the eligibility criteria and access requirements, and the source reference. Initial information was gathered through web searches and a MLOHT supporting document. The MLOHT supporting document included a partial inventory for the London-Middlesex region, compiled as an appendices to accompany the original application to create the MLOHT. Tier 4 is the full inventory of the identified programs and services in the London-Middlesex region; a short excerpt of the inventory is presented in Table 3. There were 26 providers identified, offering a total of 131 programs or services. Of these, 30 programs were open access, meaning that a patient or caregiver could access them without requiring a referral or meeting any other eligibility criteria.

**Table 3:** Tier 4, the inventory of programs and services identified in the London-Middlesex region (excerpt taken from the full inventory)

Provider	Program or Service	Eligibility and Access	Fees	Care Element Addressed	Source Reference
**Alzheimer Society**	Memory Screening	Open	No fee	Pre-Diagnosis and Primary Prevention, Maintenance and Secondary Prevention, Long-Term Care	Supporting Documents for Middlesex London Ontario Health Team
	Dementia Education	Open for people with dementia	No fee	Maintenance and Secondary Prevention, Long-Term Care, Psychosocial Support	Supporting Documents for Middlesex London Ontario Health Team
	Caregiver Support and Education	Open for caregivers of people with dementia	No fee	Maintenance and Secondary Prevention, Long-Term Care, Psychosocial Support	Supporting Documents for Middlesex London Ontario Health Team
	Powerful Tools for Caregivers	Open for caregivers of people with dementia	No fee	Maintenance and Secondary Prevention, Long-Term Care, Psychosocial Support	Program-Guide-Winter_Spring-2020_ONLINE.pdf (alzheimerlondon.ca)
	Safety Programs (Wandering Support)	Open for caregivers of people with dementia	No fee	Maintenance and Secondary Prevention, Long-Term Care, Psychosocial Support	Supporting Documents for Middlesex London Ontario Health Team
	1:1 Social Work Counseling	Open for people with dementia	No fee	Maintenance and Secondary Prevention, Long-Term Care, Psychosocial Support	Supporting Documents for Middlesex London Ontario Health Team
	Social Recreation Programs	Open for people with dementia	No fee	Maintenance and Secondary Prevention, Long-Term Care, Psychosocial Support	Supporting Documents for Middlesex London Ontario Health Team
	In-Home Recreation Program	Open for people with dementia	No fee	Maintenance and Secondary Prevention, Long-Term Care, Psychosocial Support	Supporting Documents for Middlesex London Ontario Health Team
	Behavioural Support	Open for people with dementia	No fee	Maintenance and Secondary Prevention, Long-Term Care, Psychosocial Support	Supporting Documents for Middlesex London Ontario Health Team
**Best Care**	Integrated Chronic Disease Management COPD	Suspected or confirmed COPD and family physician is part of the Best Care program	No fee	Maintenance and Secondary Prevention, Exacerbation Management, Psychosocial Support	https://www.argi.on.ca/
	Integrated Chronic Disease Management Heart Failure:	Suspected or confirmed heart failure and family physician is part of the Best Care program	No fee	Maintenance and Secondary Prevention, Exacerbation Management, Psychosocial Support	https://www.argi.on.ca/

*Notes*: Supporting documents for the Middlesex London Ontario Health Team refer to a document that accompanied the original application to create the Middlesex London Ontario Health Team. It consisted of a partial inventory of available services in the London-Middlesex Region

### Linking complexity theory to practice

This pragmatic case study links key attributes of complexity theory in its methods, results, and in considerations of future state planning. The application of complexity theory in the context of this case study is presented in Table 4.

**Table 4:** The attributes of complexity theory underpinning the methodological framework used and the interpretation of the results observed

Attributes: Complexity Theory	Application of complexity theory to this case-study
**Agents** *Individual agents work within a complex system* I**nteractions** *The agents interact exchanging information or directing the flow that the information can go*	This case-study focused on the **agents** and the main components of the health system that they act within. Involving the main **agents** in the co-creation approach facilitated the construction of a map that illustrates the relationship between the different components across the health system, identifies the elements of care within, and the **interactions** between these components (Tier 1 and 2). **Interactions** in tier 1 were displayed unidirectionally. This helped identify the navigational path between components of the health system (e.g. the patient or caregiver must interact with certain components to progress through the system). In this case-study we did not define the criteria to initiate **interaction** or the specific nature of the **interaction** but acknowledge the need to do so to facilitate future state planning.
**Non-linearity** *These interactions are not all equal and the effect of one interaction may have a larger effect*	Complexity theory acknowledges that complex systems are **non-linear** making the impact of interactions unpredictable. **Non-linearity** demands that important system outcomes be measured in the future state system performance assessment.
**Unpredictability** *Individual agents have freedom to work in ways which are not predictable, therefore it is hard to predict the effect of an intervention*	The **agents** defined across sectors in our health ecosystem act autonomously. Program volumes, the extent to which care elements are implemented, program standards and outcomes are not known across the whole health system. It is therefore hard to predict the effect of interventions across the system. **Unpredictability** supports performance and outcomes measurement at the **agent** level.
**Path dependence** *Complex systems display sensitivity to their initial conditions and therefore two comparable systems may behave differently depending on their histories*	The health care ecosystem mapping process revealed that resource availability was not uniform across the system and system components were limited by resource availability. For example primary care providers had different resource availability based on family health team and geographical location. Therefore **path dependence** will be important to consider in the redesign of the system; consideration of how components operated in the past may differentially influence intervention impact across the system.
**Self-organization** *An internal characteristic of the system to adapt intrinsically to increase system stability*	**Self-organisation** was elegantly described by primary care providers. Resource availability limited the ability to offer extended primary health care to address the psychosocial health needs for their patients. Therefore, the system **self-organised** with acute care dominating interventions. This structure enhances stability by ensuring unstable patients are prioritized. Further work should be done to identify leverage points that might foster more proactive and preventive care as well as consider how the current rules governing resource distribution contribute to present inequities and inefficiencies.
**Emergence** *The individual interactions between components of a system may produce a property of the system that is different than the sum of the overall individual agent behaviours*	The health care ecosystem map started with a “zoomed out” version of the system. The purpose was to be able to consider the **emergent system** as a whole rather than narrowing the focus, viewing only the parts or components perceived to be key to the target diseases. The aim was to encourage an understanding of the system’s functional behaviour as a web of **interactions**.
**Open system** *Undefined boundaries, the boundaries are permeable* **Diversity** *Composed of differing elements*	The health system is an **open system**, that can re-organise considerably as a result of external stimuli. Not only is the outcome of any intervention **unpredictable** but the response of the system to any outside disturbance is unknowable. In this case-study we applied disease specific constraints as a boundary setting, designed to balance the complexity associated with boundary permeability with the limitations of a simplified but workable model. We acknowledge that patients are **diverse** in terms of their health care needs and experience of the health care system. However, underpinning **diversity** and one of the drivers of these differentials are broader social, economic and contextual factors. One limitation of the current system is treatment of individuals as units of disease and not necessarily as people who themselves exist within a complex ecosystem of unequally distributed resources too. This study revealed system boundaries are permeable. It could be valuable to visualise a system where the components are more sensitive and reactive to the complexity of the biopsychosocial existence and try to identify leverage points for intervention to promote this version.

Definitions of attributes [17–21, 23]

## Discussion

### Key findings

#### Summary of the study

An interdisciplinary working group with a commitment to health system transformation used a multi-method co-creation approach to develop a current state, health care ecosystem map for a vulnerable population of patients with COPD and HF. The map identified sectoral components of a complex health care system, the elements of care required, and inventoried programs and services that deliver care to a target patient population. This process, promoted an understanding of the health system as a whole, identified gaps in the current state care elements, and established a framework for discussion. The health care ecosystem map created for the COPD and HF population, is a template that can be lateralized and adapted for other disease states and to other health systems. This work will be used to inform health system transformation and modelling.

#### Interactions

Tier 1 is a practical attempt to simplify a complex system, however, the complexity remains apparent with a multitude of touchpoints distinguished. There were only 8 touchpoints (13%) directly available to the patient. An indication that even a fully informed patient cannot navigate the health care system unaided. In contrast the primary health care level supported the largest number of touchpoints (32%) corroborating evidence of a central role in a patient’s navigation through the system [38, 39]. Primary health care has been described as the “frontdoor” of the health system with demonstrated widespread impact with investment at this level [40].

#### Care elements

The more granular ecosystem representation, Tier 2, shows the diversity of care requirements within each care level. In this collaborative depiction of the current state, primary health care is responsible for providing 34 of 77 identified care elements (44%). The volume and diversity of care elements identified in the primary health care level spanned a number of disciplines accross the full biopsychosocial continuum of care. Within this study, primary care sector participants identified particular challenges delivering and accessing the “Extended Primary Health Care” and “Social Services” elements. Importantly, not all of the primary care elements presented in the ‘current state’ diagrams are currently accessible to all patients or their primary care physicians. To the contrary, the current state system reality is that these elements are not available equitably across the system.

#### Resources

The regional inventory of programs and services, represented in Tiers 3 and 4, aligned the elements of care described in Tier 2 by health care level. The detailed program and services descriptions including eligibility criteria will help to inform a future evaluation of elements of care coverage, accessibility, and current capacity compared with actual or anticipated needs. The inventory revealed that even within the boundaries of the region, the services and programs identified were not uniformly available. Eligibility to access these services was often dependent on the patient’s primary care provider team, or geographical location; some services had attached fees. According to our participants, access and eligibility were difficult to navigate, and this was managed effectively only for a limited segment of the health system through dedicated resources for service linkage and referral (e.g., Community Support Services, Central Intake and Community Care Access Centre).

### Practical interpretation

From a practical perspective, this health care ecosystem representation is a preliminary overview of our health system to inform subsequent strategic decisions. It is recognized that interactions within complex systems are non-linear and implementing change in one part of the system can have a cascading, and sometimes exponential, effect over time [27]. Tier 1 and 2 clearly identified that vulnerable patients with COPD and/ or HF are often unable to navigate the health system independently, that primary care is a ‘gatekeeper’ access point for nearly half of the identified care elements, and that care elements in primary care are not uniformly nor equitably distributed. It could follow that implementing a change or investing resources at the primary care level may provide the most system-wide impact. Beyond the care map discussion and further to this point, in this health system nearly 90% of individuals with COPD are treated by primary care physicians with only a small minority (< 11%) treated by pulmonologists [41]. Likewise, for a Canadian cohort of over 200,000 patients with HF admitted to hospital, only 17% were treated by a cardiologist [42]. Additionally the literature supports that enhanced primary care, with a focus on disease prevention and self-management, would deliver a reduction in emergency department visits and hospital admissions [7, 8, 11, 12].

A core principle of the OHT transformation strategy has been the creation of care coordinators or patient navigators to guide a patient through the system [3]. Implementing patient navigatiors in primary care is not a novel concept [38, 39, 43]. In a review of 34 studies with a patient navigation program anchored in primary care, positive outcomes (mainly descriptive) were reported for patients, providers, and the health care system [38]. In this case study, primary care sector participants felt it was not realistic, within a current or future state health system, for a solo primary care provider to deliver all the identified elements of care without system navigation and interdisciplinary support for their patients.

The mapping process laid bare the need to consider a system navigation role embedded within primary care. However, there is an interesting caveat to this and that is patient navigation can only work if there are places to navigate to and the concept of “nowhere to navigate” is most frequently encountered for patients living in rural environments [44, 45]. Tier 2 presents a seemingly comprehensive array of care elements and yet as previously discussed they are not uniformly distributed. Likewise Tier 4 identifies programs and services available regionally but we need to consider the capacity of these services, wait times, access requirements, and regional variability. Which poses the question, is it an increase in services availability that is required or is it the coordination of access to them or perhaps a combination of both? The health care ecosystem map cannot be used to answer these questions but provides a valuable discussion board, that emphasizes the complexities that need robust consideration before generating innovative future implementation strategies.

### Limitations

This research has several limitations. While a multi-methods approach was utilised to maximise co-creation of our health care ecosystem maps, response proportion declined throughout the process. A high proportion, (77%) of invitees attended a focus group with 5 of the 6 identified sector groups represented. Response to the document review (Steps 4 and 5) was 33 and 8% respectively. Plausible explanation for this lower response is that non-responders felt they had already contributed to the process through focus groups, or possibly they were aware that other representatives from their sector had responded negating their obligation to respond. The lowest response proportion was for the Tier 4 inventory (8%), potentially because non-responders had no corrections or additions or that the burden of a detailed review was too great. To ensure the outcome of Tier 4 is sufficient, this work will be re-assigned in the next phase of our development work.

Another limitation was the potential bias of the Sub-Group in creating the initial base model (Step 1). While the Sub-Group did represent a number of the identified sectors, not all members of the Working Group were present at the “blank slate” stage of development. We balanced the loss of full sector involvement in Step 1 with the benefits of smaller group dynamics such as increased productivity and greater resource efficiency to facilitate and coordinate the phase 1 process [29]. We acknowledge that the ecosystem maps represent how the system is perceived to work from the perspective of the co-creation team. By using a co-creation approach with expertise spanning the breadth of the health system we have, by design, attempted to narrow the gap between actual system structure / function and perceived system structure / function.

Finally, contribution from the Patient, Client and Care Partner Council achieved a non-health professional perspective, however, experience of the health system was not specific to COPD and HF. In addition feedback was aggregated with no delineation of patient input. We believe for the phase 1 objectives this group provided adequate representation. For phase 2 robust, disease specific, patient and caregiver co-creation is planned.

### Theoretical contribution

“Systems theory has been challenged in the recent literature due to its perceived disconnection from today’s research and practice demands” ([23], p.1). In this case study we connect the attributes of complexity theory to the development of the health care ecosystem map, identify where attributes may have affected the current ecosystem and identify areas where understanding complexity theory can guide future state planning. Presenting the attributes of complex system theory in the context of a pragmatic case-study may be useful to the “non-theorist” scholar-practitioner and health system planners.

### Future work

The next phase for the MLOHT working group utilises the phase 1 learning presented in this manuscript, and leverages established collaborative relationships, to: (i) explore the usefulness and feasibility of a health system model and notation [34] to predict system resource requirements, and (ii) inform implementation of a redesigned care delivery plan to be tested on 2000–3000 recruited adults with a primary diagnosis of advanced COPD or HF. Outcomes will be measured using a health equity-driven quadruple aim framework to assess the impacts of the system redesign.

## Conclusions

A series of visual representations (health care ecosystem map) were developed using a co-creation approach to support the early phase of health system transformation. The map confronted the system in it’s complexity, unpacking the web of interconnectivity between and within the different sectors reinforced the idea that implementing a change in one area of the system can resonate throughout the whole system. There is an overwhelming body of literature on complex system methodology, most begin by looking at the structure of the system, its components, and the interactions within. This case study documented a practical methodology for this first step, and presented a template health care ecosystem map. Academically, this case study provides a pragmatic illustration of complex system methodologies that will support frontline innovators to bridge the gap between theoretical modelling and implementation.

## Supplementary Information

**Additional file 1.** Step 1 The Base Model.

**Additional file 2.** Step 4 questionnaires and feedback from 12 reviewers.

**Additional file 3.**

## Data Availability

All data generated or analysed during the current study are included in this published article and its supplementary information files. Editable versions of the health care ecosystem map templates can be requested from the author of correspondence.

## Abbreviations

COPD: Chronic Obstructive Pulmonary Disease
HF: Heart Failure
OHT: Ontario Health Team
MLOHT: Middlesex London Ontario Health Team
