# Supplementary material for: Confronting complexity and supporting transformation through health systems mapping: a case study
Source: BMC Health Serv Res. 2021 Oct 23;21:1146. doi: 10.1186/s12913-021-07168-8 (PMC8540206; doi:10.1186/s12913-021-07168-8)

**Additional File 1 Step 1 The Base Model**

**Tier 1**
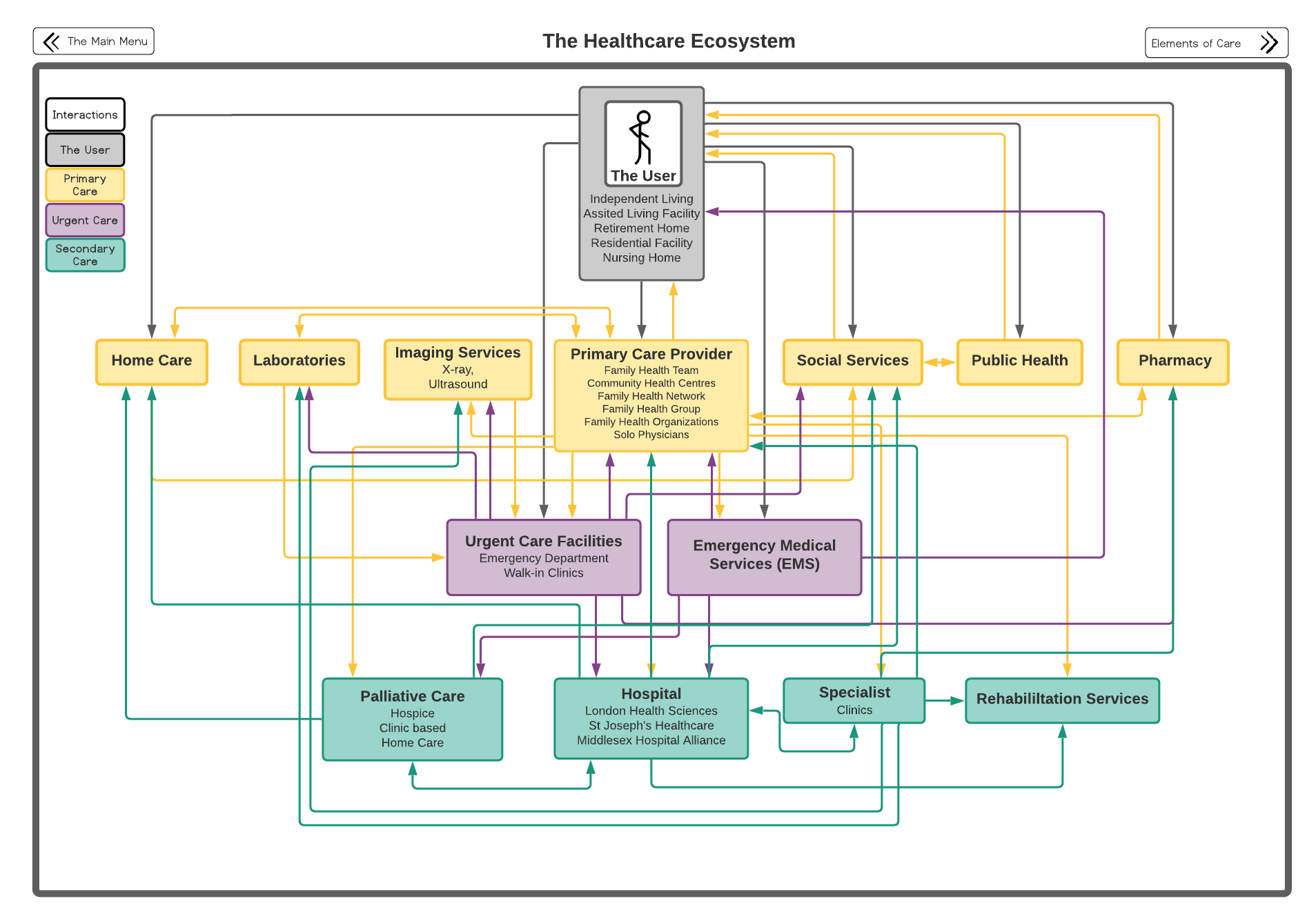


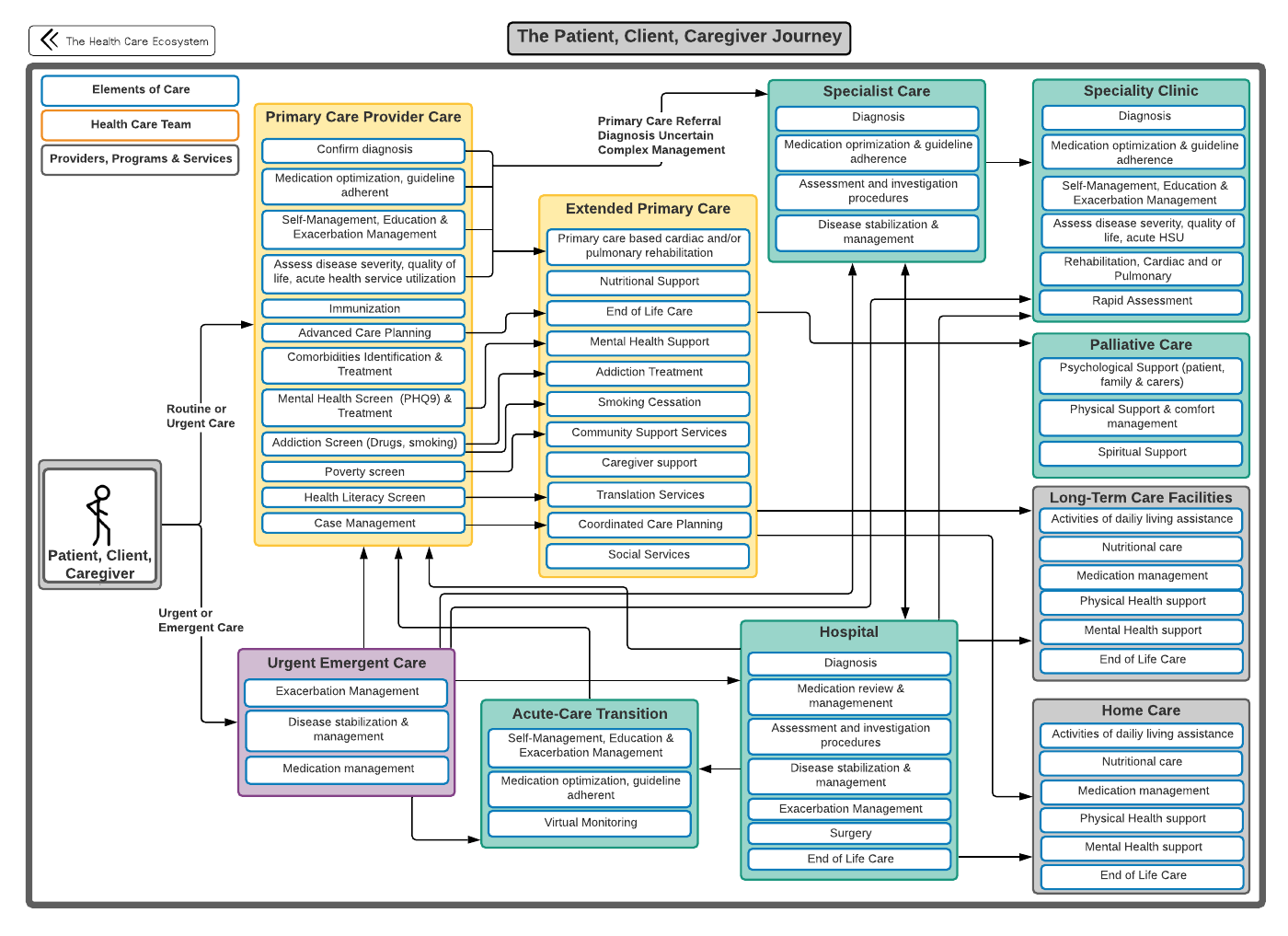
**Tier 2**

**Tier**
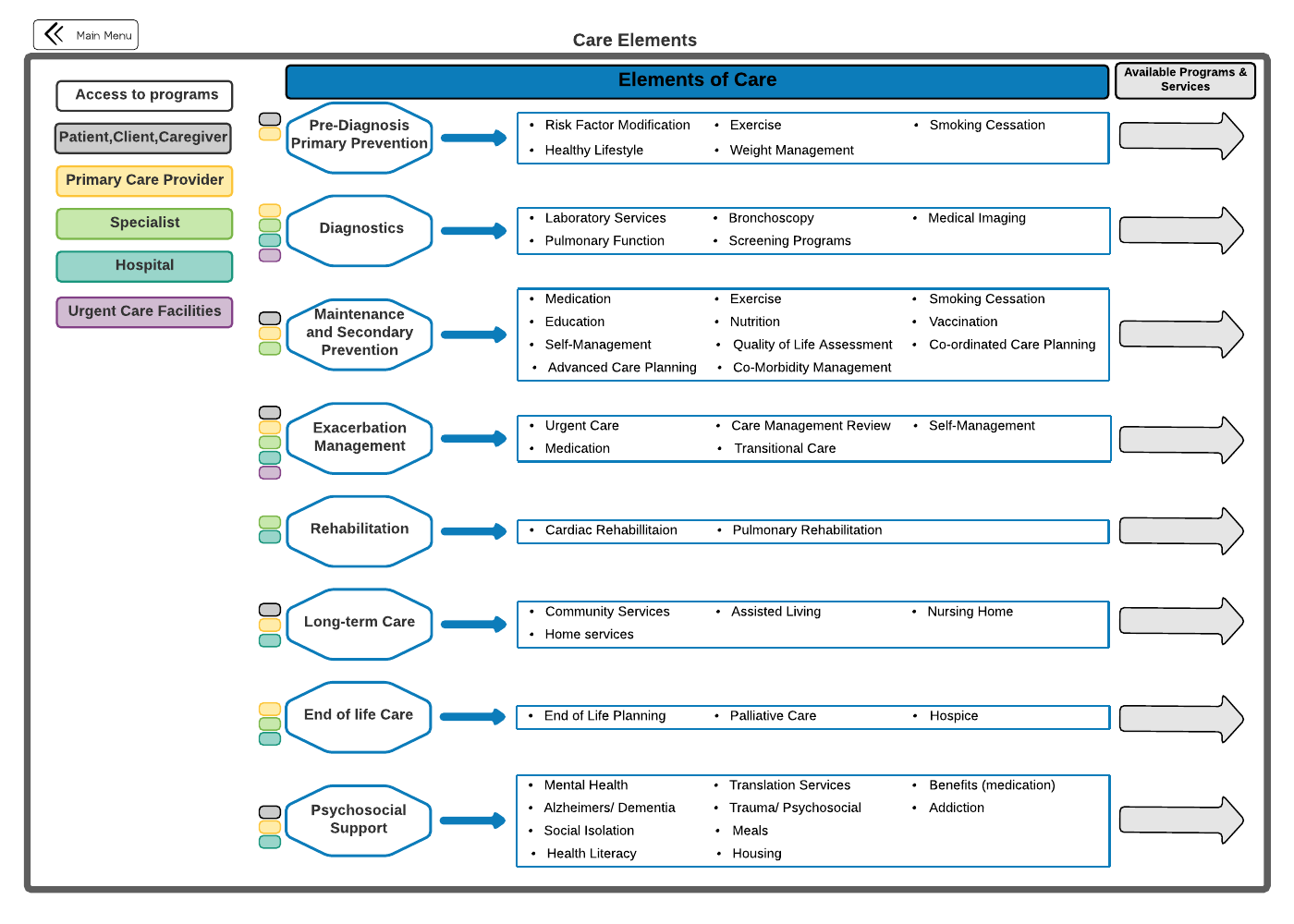
**3**

**Tier 4 Example, of the Pre-diagnosis & Primary Prevention Programs and services inventory (from Tier 3). Not shown for all elements of care categories.**


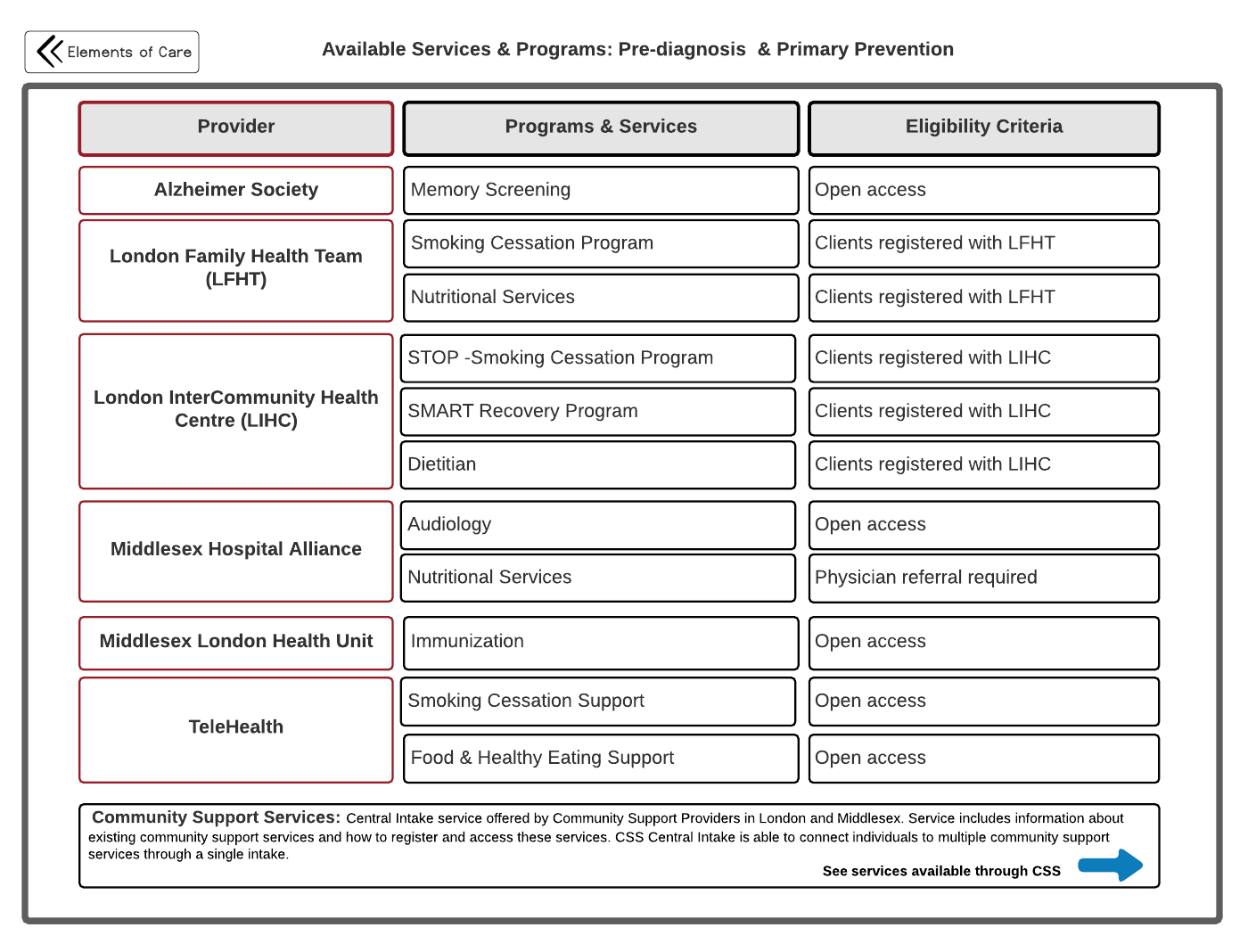

Supplement: Supplementary file 1 — Additional file 1. Step 1 The Base Model. [file 12913_2021_7168_MOESM1_ESM.docx]
