# Supplementary material for: Confronting complexity and supporting transformation through health systems mapping: a case study
Source: BMC Health Serv Res. 2021 Oct 23;21:1146. doi: 10.1186/s12913-021-07168-8 (PMC8540206; doi:10.1186/s12913-021-07168-8)

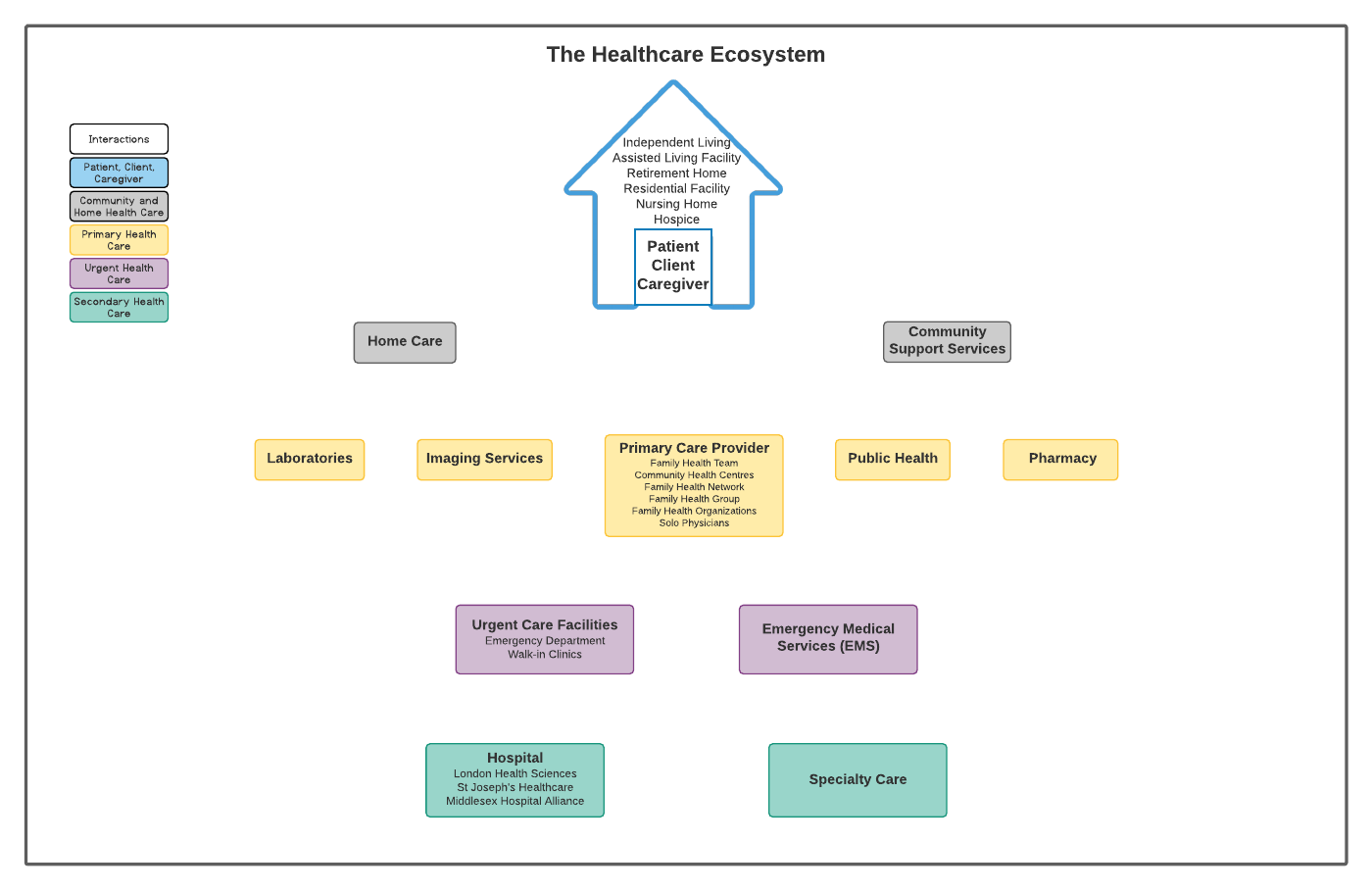
**Additional File 2 Step 4 questionnaires and feedback from 12 reviewers**

| **Questions:** |
| --- |
| **Do we have the correct key components of the healthcare system (are the boxes correct)? (If no please specify)** |
| - I might add all the private paid for services (paramedical) as well as true grass roots community (like churches and associations) as well as provimcial/federal- **Reviewer 1** - Confusion about content, Patient & Caregiver...residential facility vs. retirement home?? and other terms. Definition of public health?? Speciality care??- **Reviewer 2-5** - Other things to consider her: Government supports (ODSP, Ontario Works), Correctional Services, Mental Health Organizations, Adjunctive community supports – physiotherapy, occupational therapy, social work; - **Reviewer 6** - Looks good – **Reviewer 7** - Yes- **Reviewer 9** - Do we want to include ‘other congregate settings’ inside the house (e.g. shelter, group home)? Is Public Health reflected appropriately here, as Primary Care? Just a question - best to check with Public Health on this one :) Have we intentionally removed Social Services because this mapping is specific to Healthcare Ecosystem Mapping? Where is Mental Health & Addictions? I know that the LTCH sector is reflected inside the house but do we also need a unique box for it? (it is accessed via LHIN Home Care not directly by patients/clients/caregivers)- **Reviewer 11**   **Note:** Reviewers 2-5 completed the questionnaire as a group. |


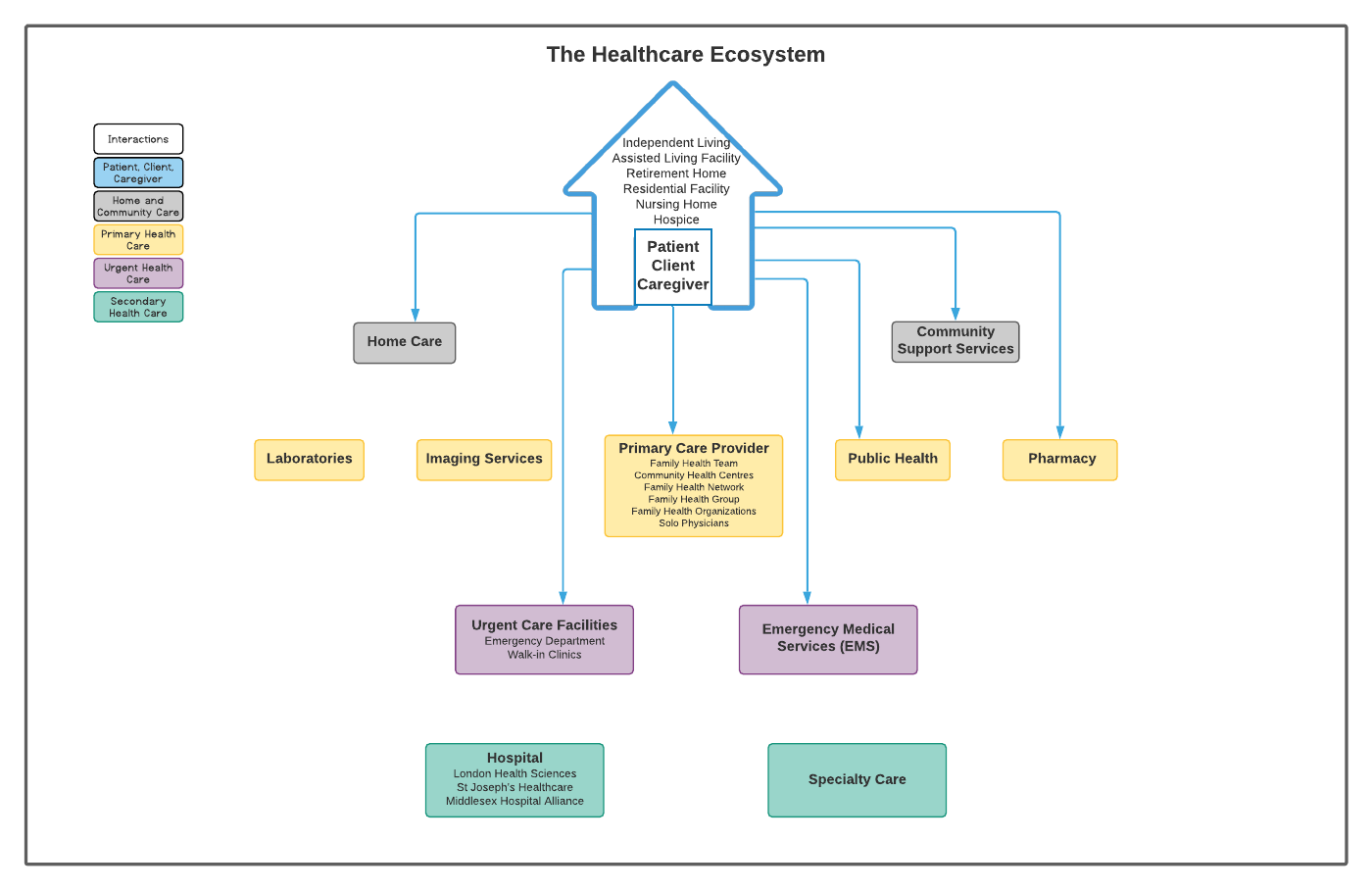


| **Questions:** |
| --- |
| **The blue arrows show the patient or caregiver pathway touch points, the components of the healthcare system that they can directly interact with. Do you think these are correct? (if no please specify)** |
| - I think that highlighting the reality of unrostered patients would be useful here- **Reviewer 1** - Directionality of arrows indicative of information flow?- **Reviewer 2-5** - I would suggest that some of these arrows are bidirectional as care can be initiated (i.e. a patient is discharged and given IV Abx, a patient then is being contacted and receiving home care). I would also suggest CSS and Public Health may reach out to patients directly. Lastly, this assumes healthcare delivery in the traditional mechanism of a healthcare transaction at a distinct point in time. What about when we send out a preventative care blast from primary care to prompt someone to seek care or change a habit..- **Reviewer 6** - Accurate for CSS**- Reviewer 7** - Once connected to a specialty service ie cardiology/ respirology palliative care would patients not have direct access to the specialty service?- **Reviewer 8** - Yes- **Reviewer 9** - Just to check – this means that the patient/client/caregiver can’t initially directly connect with hospital inpatient or outpatient or specialty care but they can once connected, right? **– Reviewer 11** |


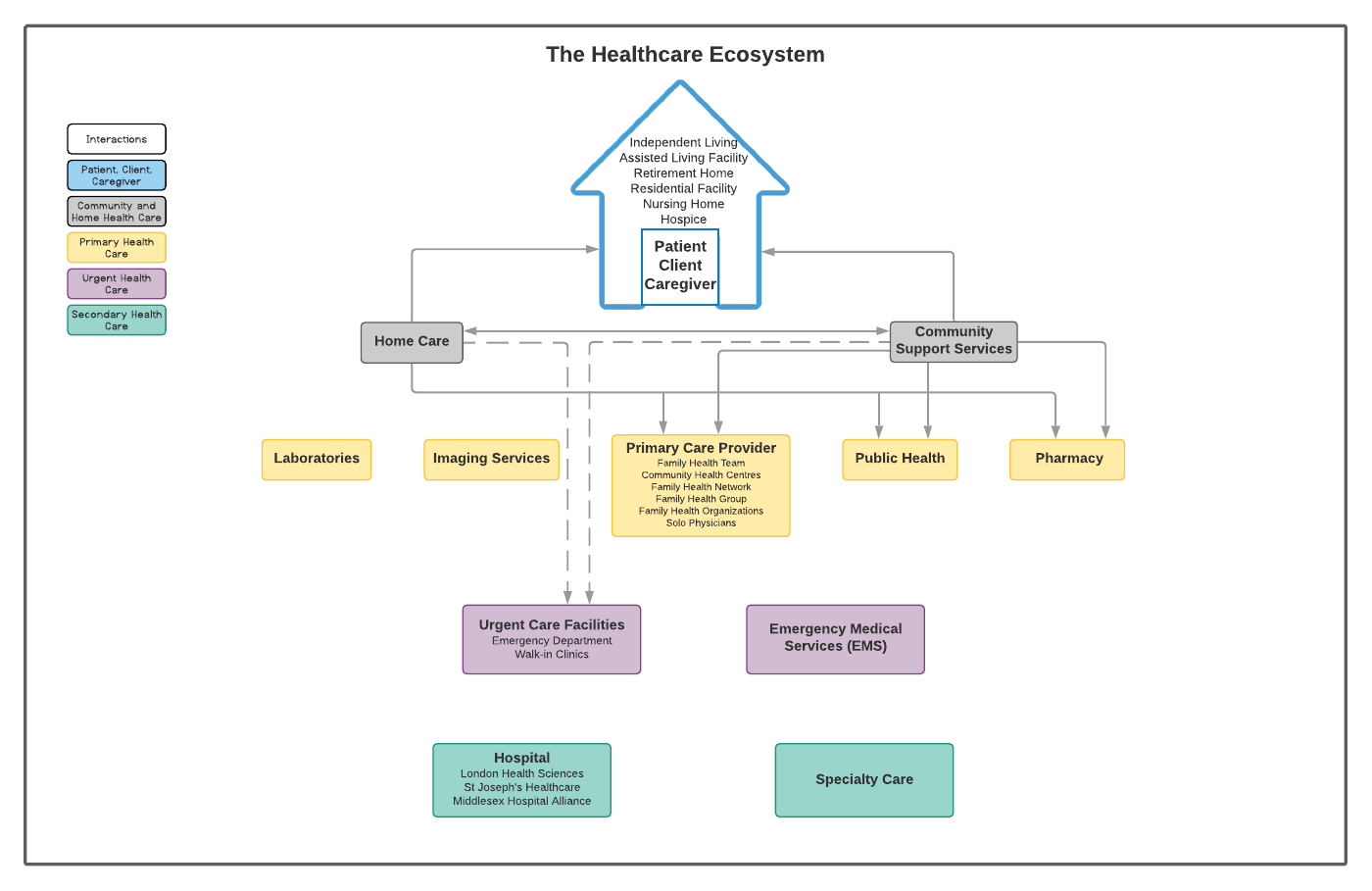


| **Questions:** |
| --- |
| **The grey arrows show the pathway to touch points from Community and Home Health Care, the components of the healthcare system that they directly interact with. Do you think these are correct? (if no please specify)** |
| - Direction of arrows re: community care to primary care??? Are arrows indicative of information flow?- **Reviewer 2-5** - I think that the homecare to primary care arrow should only be solid when there is one solid communication line between primary care and home care. If there are multiple orgs or providers they should be considered as discontinuous care. I think this slide needs a bit more work and consideration/ application of clinical cases to directly inform the connections as they exist today. If the goal of this is to provide an idea of future state connections than we need to consider a web – whereby if there is one change or alteration in a patients care that change is reflected across the system in near real time. – **Reviewer 6** - Yes for CSS -**Reviewer 7** - Are these arrows intended to show the direct connection between the patient/client/caregiver supported by Home Care and/or CSS and other services OR the connections between Home Care and CSS service providers and other services? I don’t understand the Public Health piece very well. – **Reviewer 11** |
| **The dotted grey lines are ones we are unsure of. Would Home Care or Community Support Services transport a patient to an Urgent Care Facility directly or is the route through EMS? (please specify)** |
| - CSS would not transport to ED, but could potentially provide transportation to a walk-in clinic – medical appoinments = yes; emergency = no – **Reviewer 7** - Don’t know the answer- **Reviewer 2-5** - Probably not but they may assist in transporting a patient to a specialty appointment. Ie voyager could be used to transport a patient to a cardiology appointment if it was appropriate. However, a visiting nurse from homecare could visit a patient and see that the patient was an distress and send them to the emergency department or call 911**- Reviewer 8** |


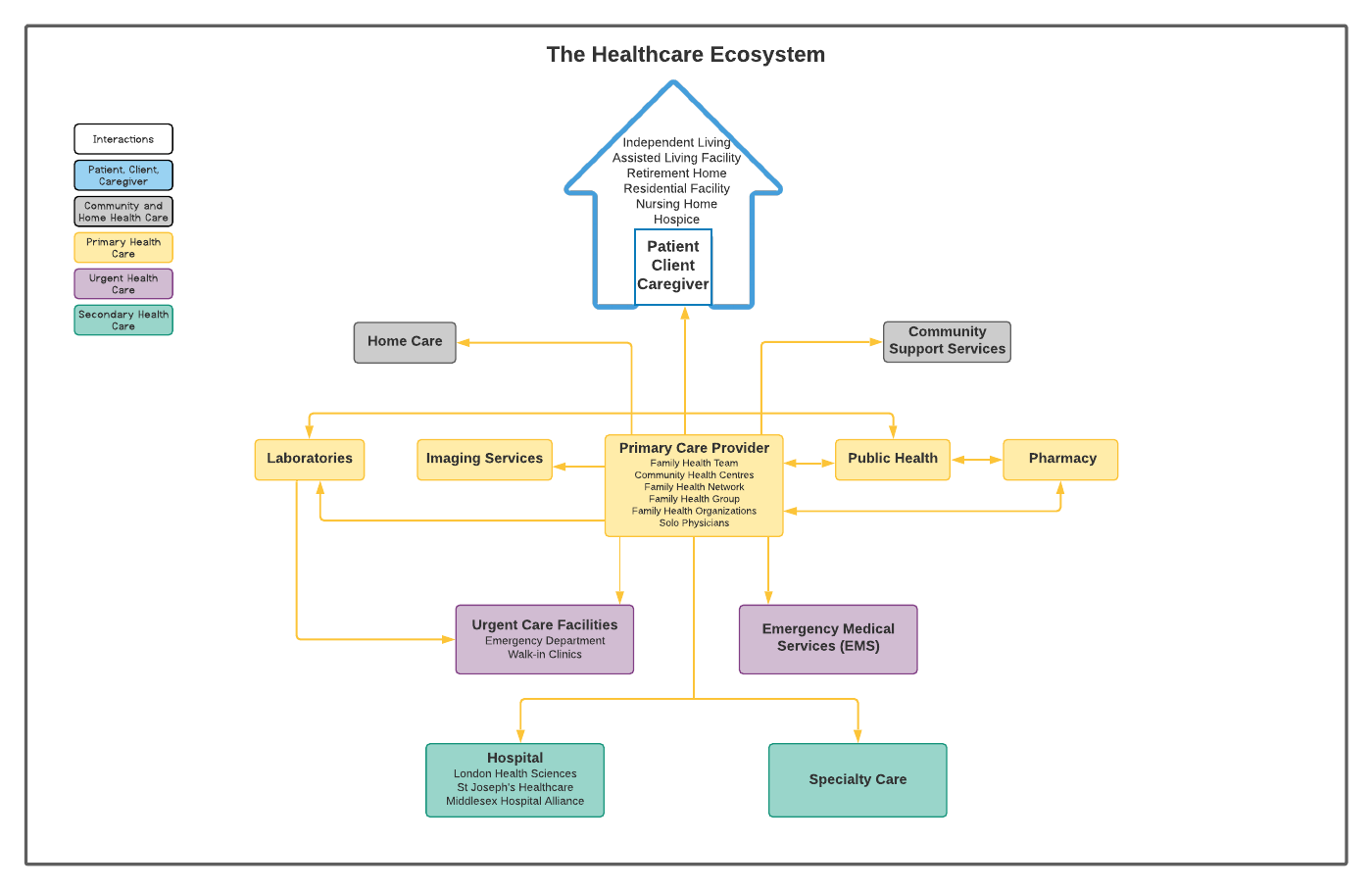


| **Questions:** |
| --- |
| **The yellow arrows show the pathway touch points from Primary Health Care, the components of the health care system that they directly interact with. Do you think the ones shown are correct? (if no please specify)** |
| - Care coordination seems to be the way that the “system” hangs together….might be interesting to map them into this – there are multiple layers and how are they organized either in support of this model or in opposition to this working?- **Reviewer 1** - Direction of arrows...see above comment- **Reviewer 2-5** - I don’t think patients go from labs to urgent care facilities but I could be wrong here..I also think that some of the connections here are again multi-faceted instead of linear but I think the overall connections are appropriate- **Reviewer 6** - Yes- **Reviewer 7** - Yes- **Reviewer 9** |


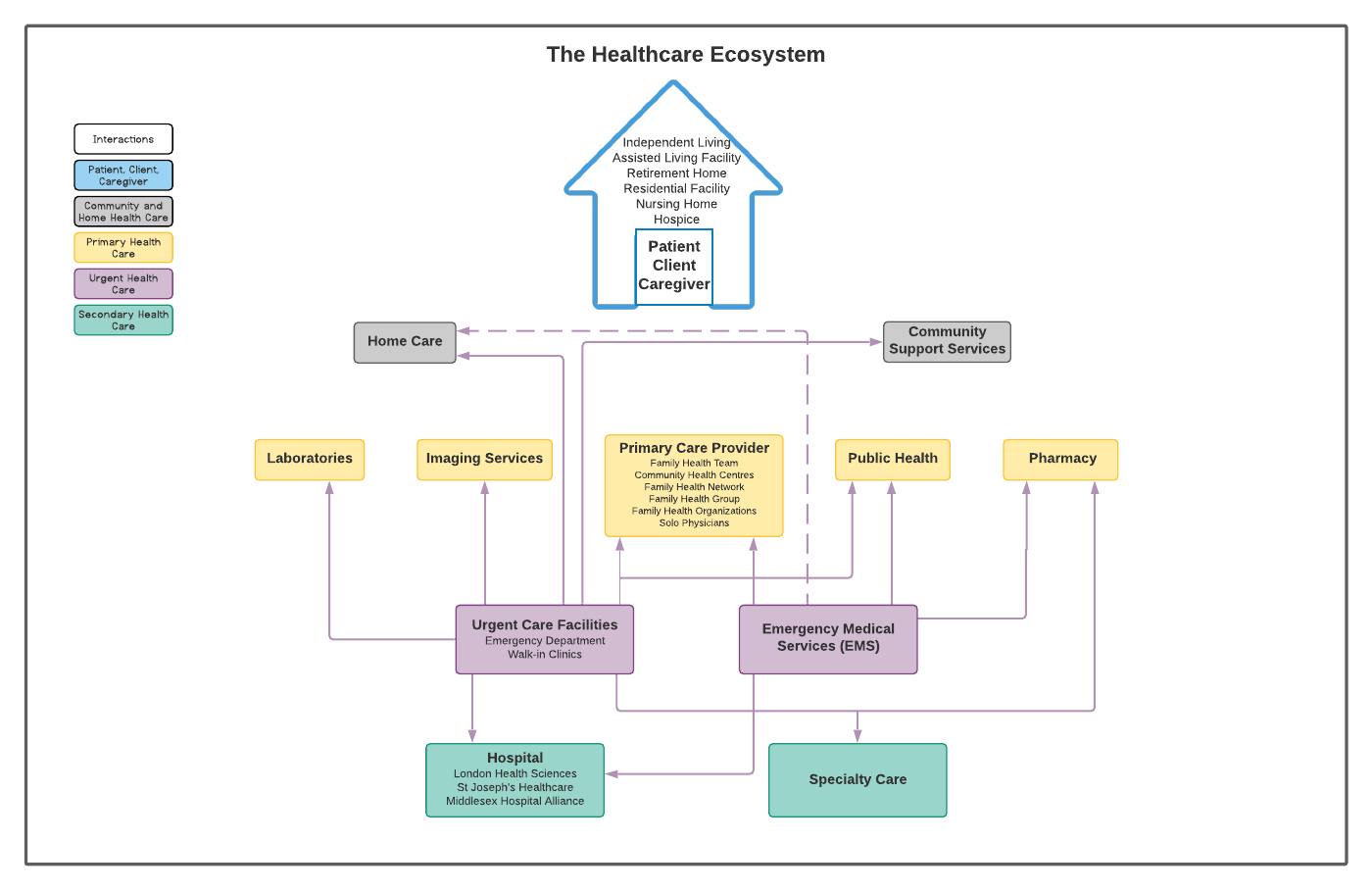


| **Questions:** |
| --- |
| **The purple arrows show the pathway touch points from Urgent Health Care, the components of the health care system that they directly interact with. Do you think the ones shown are correct? (if no please specify)** |
| - This is where the biggest breakdowns occur – post this experience…I think adding the how behind the arrows might be useful…hospital back to primary care…primary care to EMS…how….- **Reviewer 1** - No link to patient/care partner????- **Reviewer 2-5** - Yes- **Reviewer 7** - Yes- **Reviewer 9** |
| **At present we have Paramedicine under EMS. Where do you think Paramedicine fits into the ecosystem and should Paramedicine link to Home care and Community Support Services (the dotted line)?** |
| - We should use them more intelligently wherever the best augment the system- **Reviewer 1** - Not sure..what is paramedince? How is it accessed?- **Reviewer 2-5** - I think community paramedicine is a primary care service which works to provide primary care in the community. I would suggest that we should create a triad structure of primary care services that include – primary care/ Community paramedicine / CSS + Homecare. These services would need to function as one integrated care unit to deliver good, preventative upstream care. I would like to see this change reflected in the physical organization and positioning of those in the diagram. IF you need further clarification let me know! – **Reviewer 6** - Community paramedicine should have link to home care and CSS – testing CSS Bundled services for supported discharge, which includes Community Paramedicine as a component – **Reviewer 7** - Yes- **Reviewer 9** |


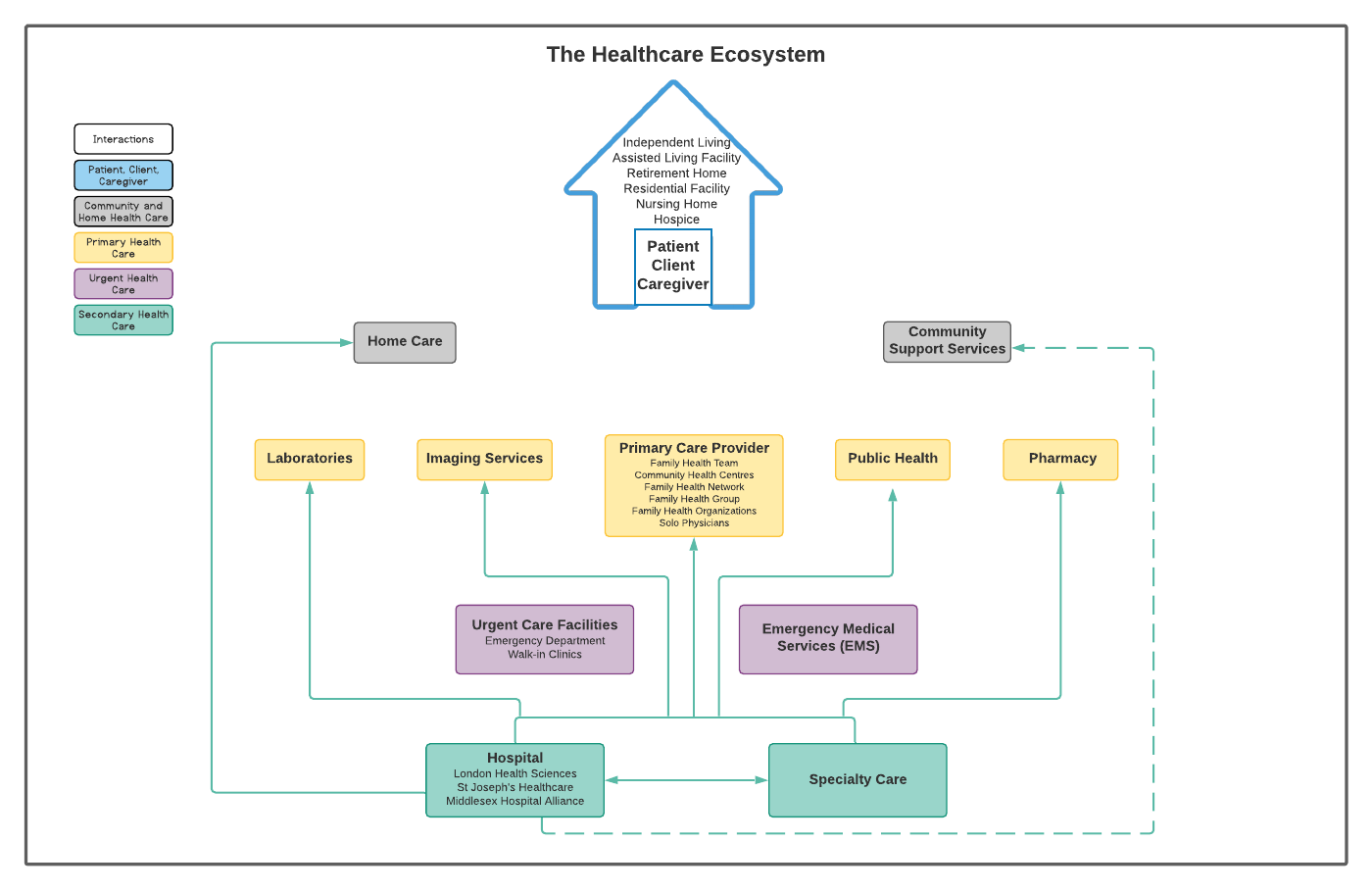


| **Questions:** |
| --- |
| **The green arrows show the pathway touch points from Secondary Health Care, the components of the health care system that they directly interact with. Do you think the ones shown are correct? (If no please specify)** |
| - The hospital doesn’t interface with homecare – generally the patient is given a piece of paper with a number. I think that exploring from the patients POV how THEY engage/CAN engage/Have HAD to engage would be telling. I agree with this chart – the patient is an island with no obvious breadcrumb to pick back up if there are gaps/questions/concerns- **Reviewer 1** - No link to patient/care giver???- **Reviewer 2-5** - I think most lab and imaging work In secondary health care take place within the hospital or specialty offices. These are usually distinct imaging and labs from what primary care has access too. It would also be important to demonstrate that primary care has access to some of those secondary labs and imaging centres but not all. There is a tier of diagnostics and labs we are missing. **– Reviewer 6** - Yes- **Reviewer 9** |
| **Does the hospital refer directly to Community Support Services? (please specify)** |
| - Never been my experience – but I have gotten a photocopied paper of possible services**- Reviewer 1** - Yes...on discharge, referral to social workers, public health well baby visits, meals on wheels, PHSS, VON, for example**- Reviewer 2-5** - Not that I’m aware of. **– Reviewer 6** - Yes – we are actually seeing an increase in these direct referrals- **Reviewer 7** |


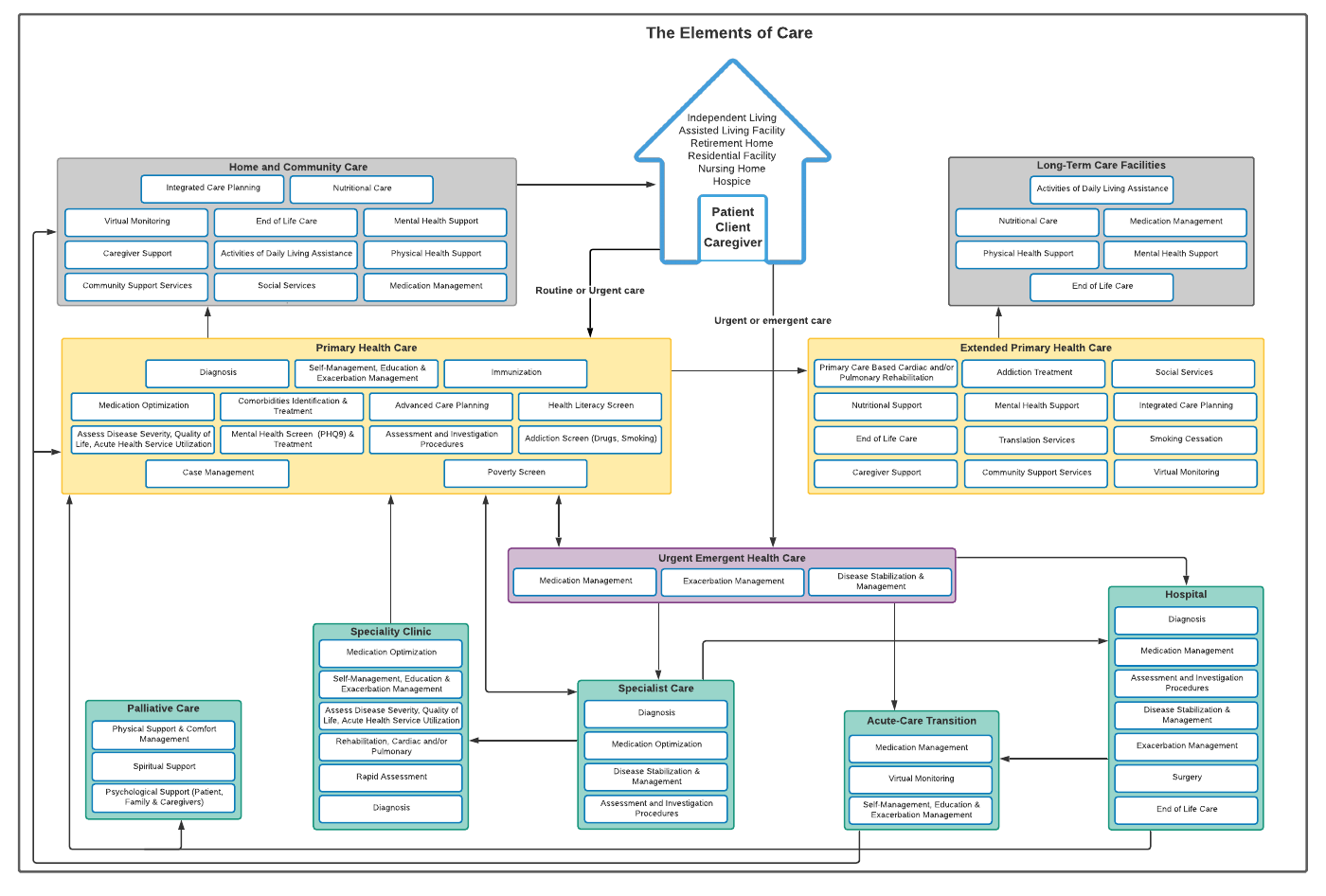


| **Questions:** |
| --- |
| **This visual representation shows the pathway of the patient and caregiver through the health system with a focus on COPD or heart failure. It shows the different elements of care that a patient may potentially receive at each level of care. Please review these elements of care, do you think they are correct? (if no please specify)** |
| - Too complex diagram. Who are these people? e.g. Urgent Emergent Health Care?? Who is represented? Language is too complex..eg.. what is medication optimization? Exacerbation management? – **Reviewer 2-5** - Yes- **Reviewer 7** - Yes-**Reviewer 9** - Our wording on page 1 should match this page re: Home & Community Care (on page 1, called “Community and Home Health Care”   Long-Term Care appears here, so thinking that it should appear on page 1 as well  Social Services reappears here but isn’t shown on page 1  I’m not sure if all of the Home and Community Care elements grouped into one bucket will resonate for all or not (will let people from the various sectors advise us, but just flagging) – good to See Mental Health here but again, not sure if it should be positioned under “Home and Community Care” – it should be Mental Health & Addiction  Where did Public Health go? I was kind of confused about how it fit but not sure why it shows up on all pages except for this one – there is a role of Public Health re: Immunizations - I think I need someone to talk me through this piece:)  Not sure if I understand “Health Literacy Screen” – is this asking patients/clients/caregivers about their language of comfort?  May want to double check with our MHA partners re: Mental Health ‘Support’ and Addiction ‘Treatment’ – I wonder if it is ‘support’ for both in that we may be meeting people where they are at and supporting them with their addiction vs. treating?  What does Community Support Services under Secondary Primary Care mean?  I see “Disease Management” under Urgent Emergent Health Care; under Primary Care, is Disease Management captured under “Comorbidities Identification and Treatment”?  Why do we have Acute-Care Transition but no other transitions included?  I don’t understand why we have Specialty Clinic separated from Specialist Care – why not just Specialty Care all together?  Why do we have Palliative Care on its own? – **Reviewer 11** |
| The arrows show the patient pathway through the system whether they enter through Primary Care or Urgent Emergent Care. Do you think these pathways are correct? |
| - I don’t know – have no navigated this myself for COPD- **Reviewer 1** - Too complicated to understand. No lived experience to comment with authority- **Reviewer 2-5** - I think they accurately represent our current. I don’t think I would propose any substantive changes but when we begin to enroll patients into a year 1 population work I would like to explore and map out how actual patient journeys map onto this! **– Reviewer 6** - Yes- **Reviewer 7** - Patient can be refereed to Palliative care by primary care, Specialty Care and specialty clinic and Hospital   Just as a note Palliative care may be provided by the primary care physician or by the specialty PC team. Patients in later stages of their illness are often referred to the palliative care team by their primary care physician to take over care as the family doctor does not do home visits. In other cases palliative care may do a shared cared model with the primary care physician or just do a one off consult. As a specialty service We maybe involved at variouse stages of the patients illness. We may follow them early on as out patient consult service. They may also see PC in hospital.  In the Palliative care box stead of saying “Physical support and comfort management” maybe say “ Pain and symptom management”  Given that primary should be doing some of the palliative care I wonder if in the primary health care block we should also add “pain and symptom management and psychological support “ – **Reviewer 8**   - Primary care can refer directly to Pulmonary rehab under “specialty clinic”- **Reviewer 9**   Additional Comments:  I wanted to just highlight the language around this document, as below:   - Local Health Integration Network (Home and Community Care) versus Community Care Access Centre. - Care Coordination (Allied Health) versus  Clinical Coordinator (RN, RRN, NP). - Role delineation to coordinate care, provide :system navigation, continuity of care, linking to community supports or escalating through MRP or EMS. - Coordination in all transitional touch points (phone, in home, virtual, ED, Hospital, RH to LTC). - **Reviewer 12** |

**Reviewer 10**


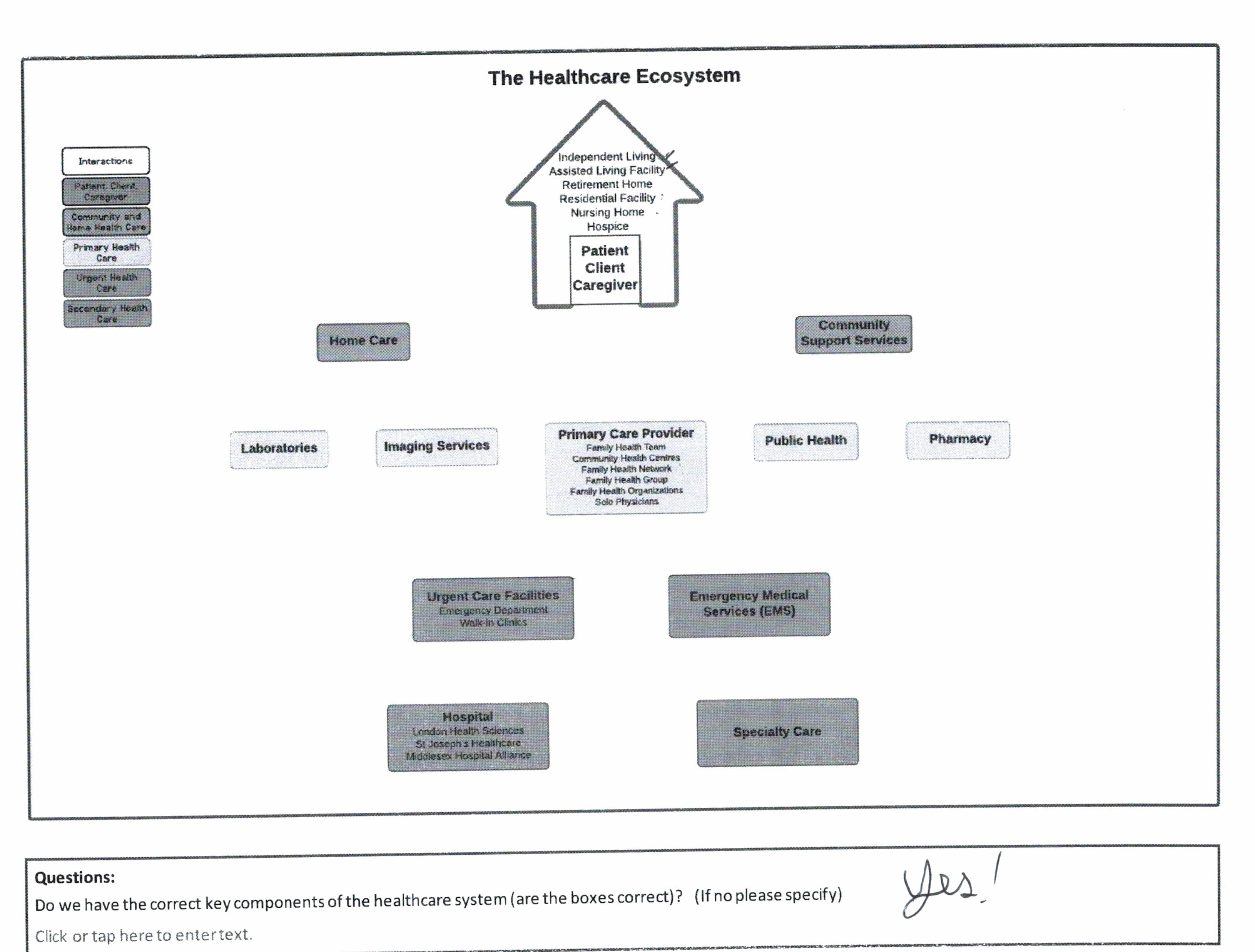


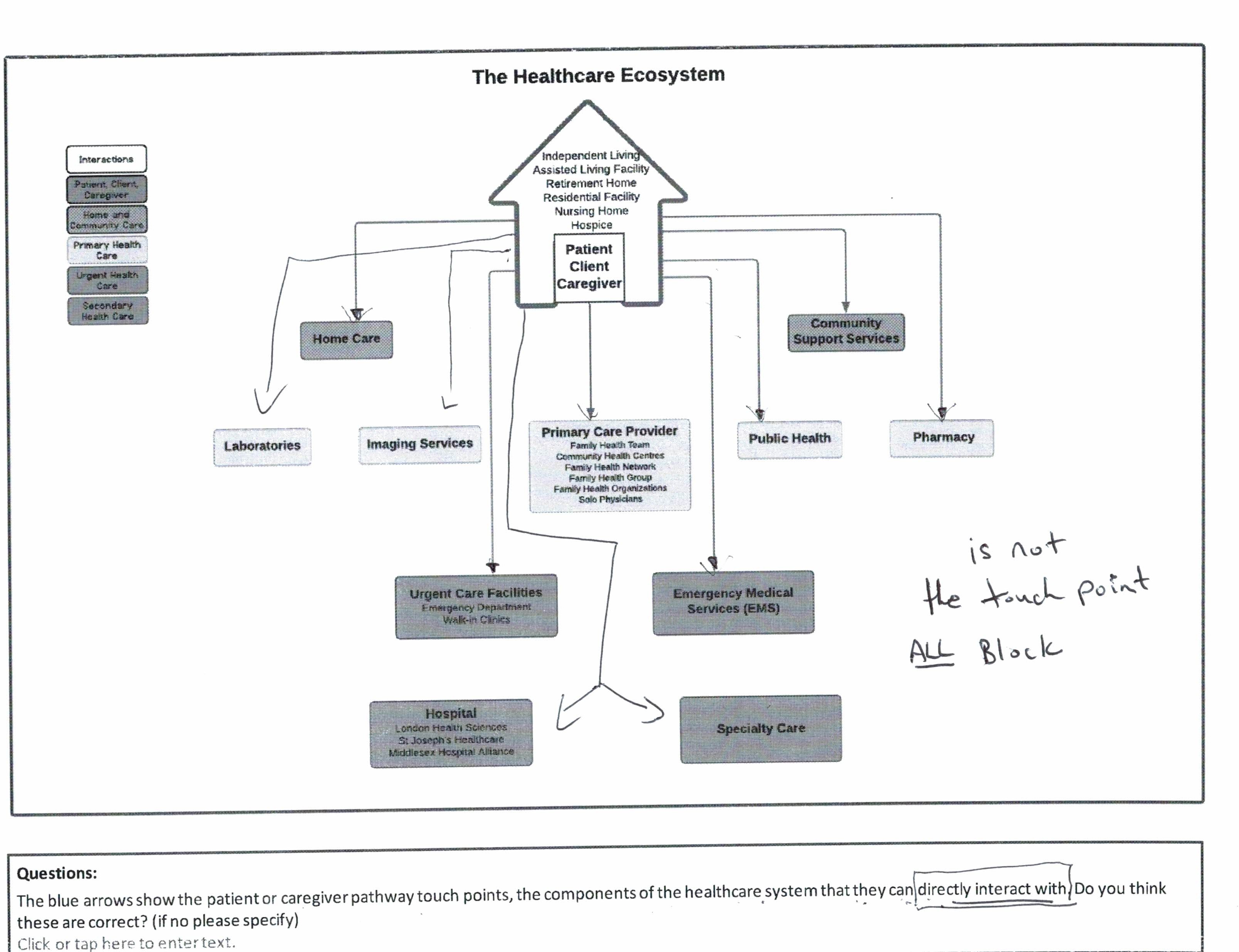


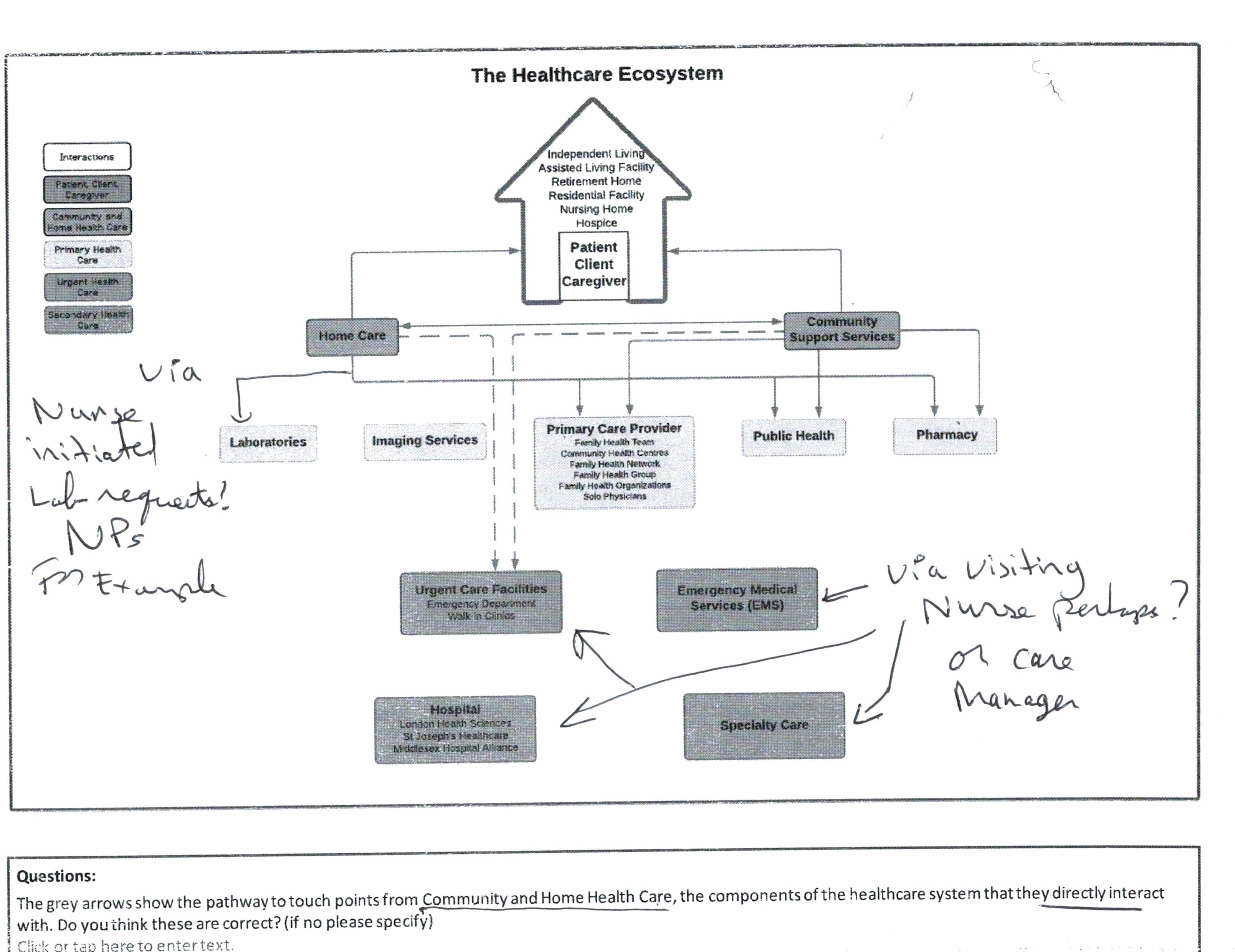


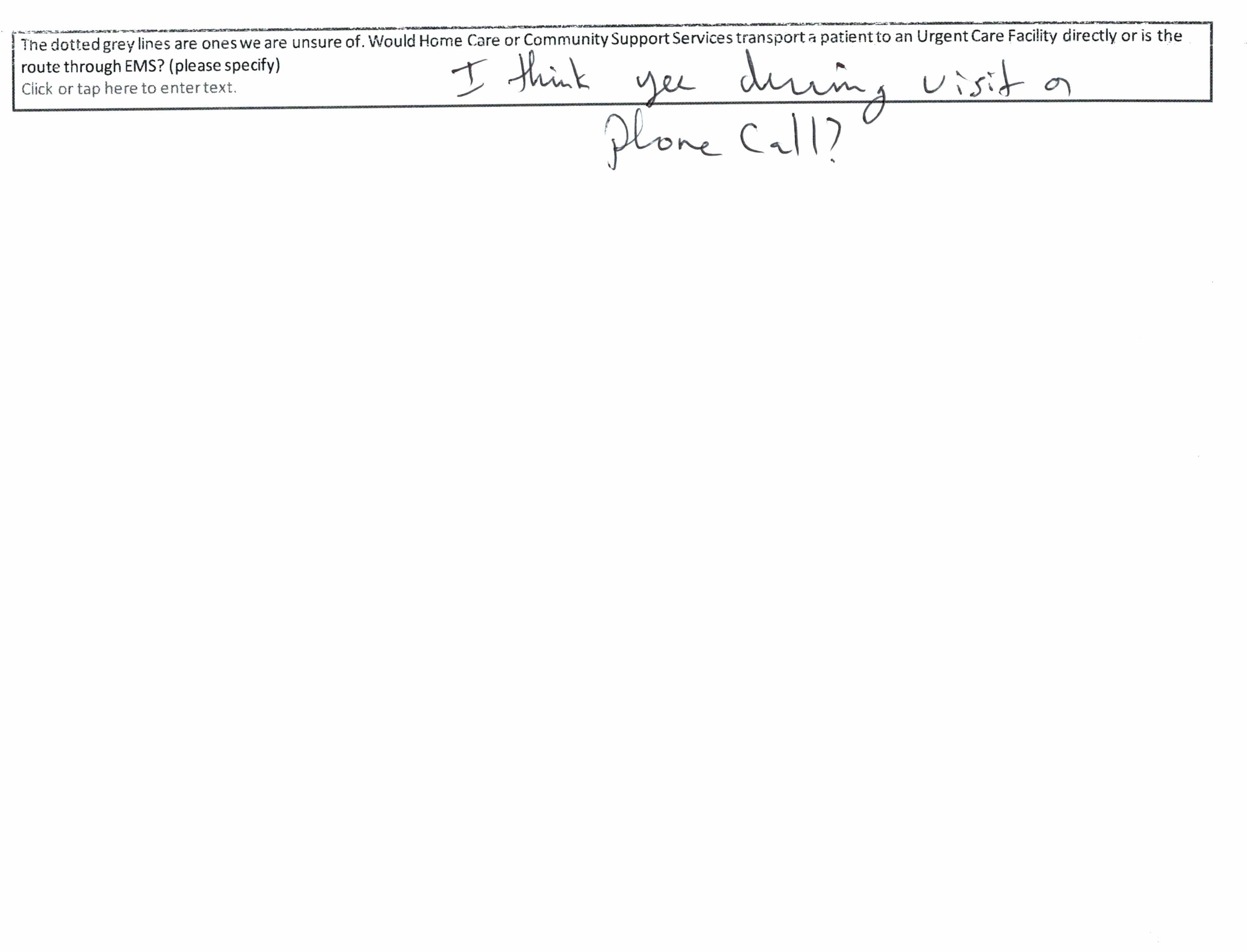


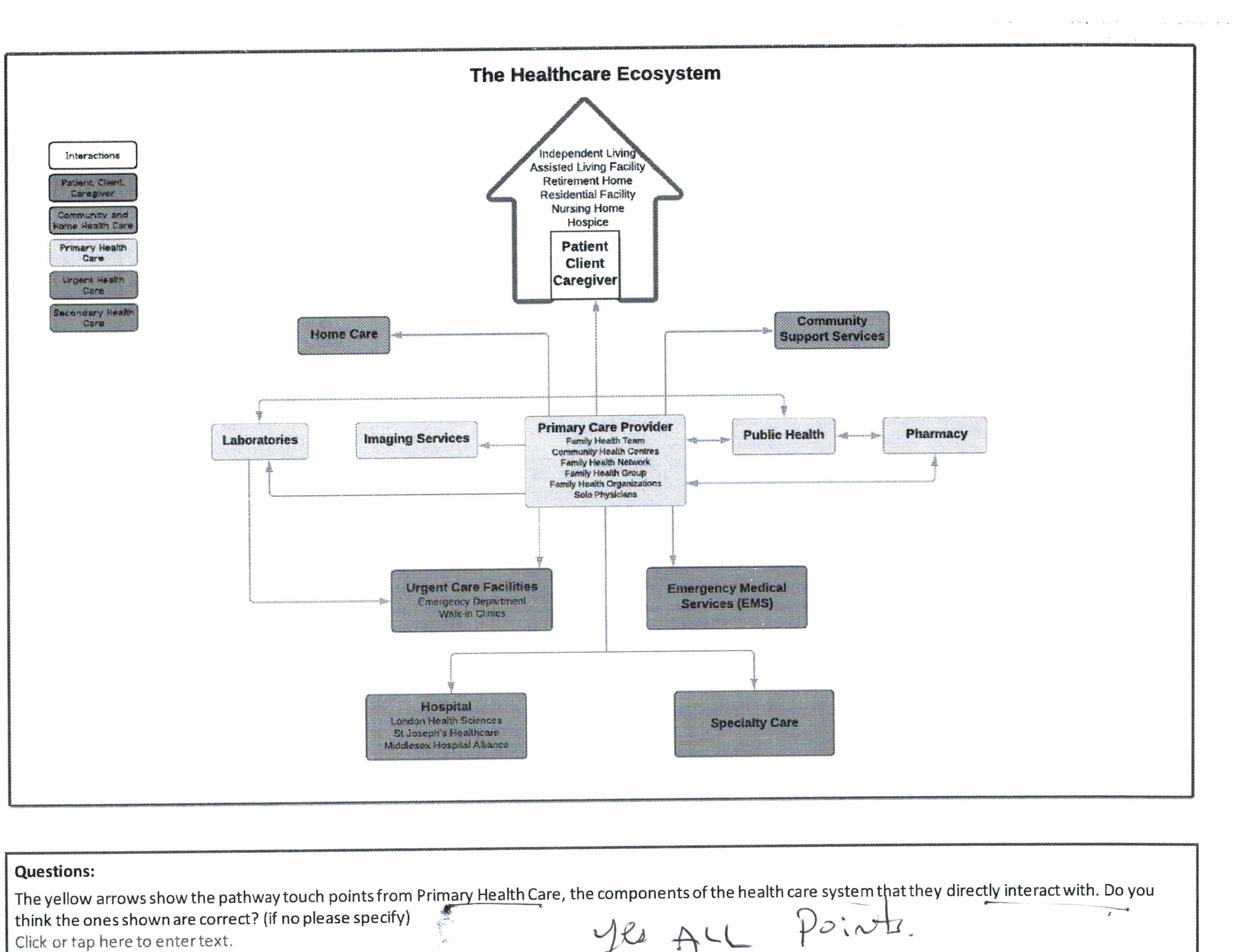


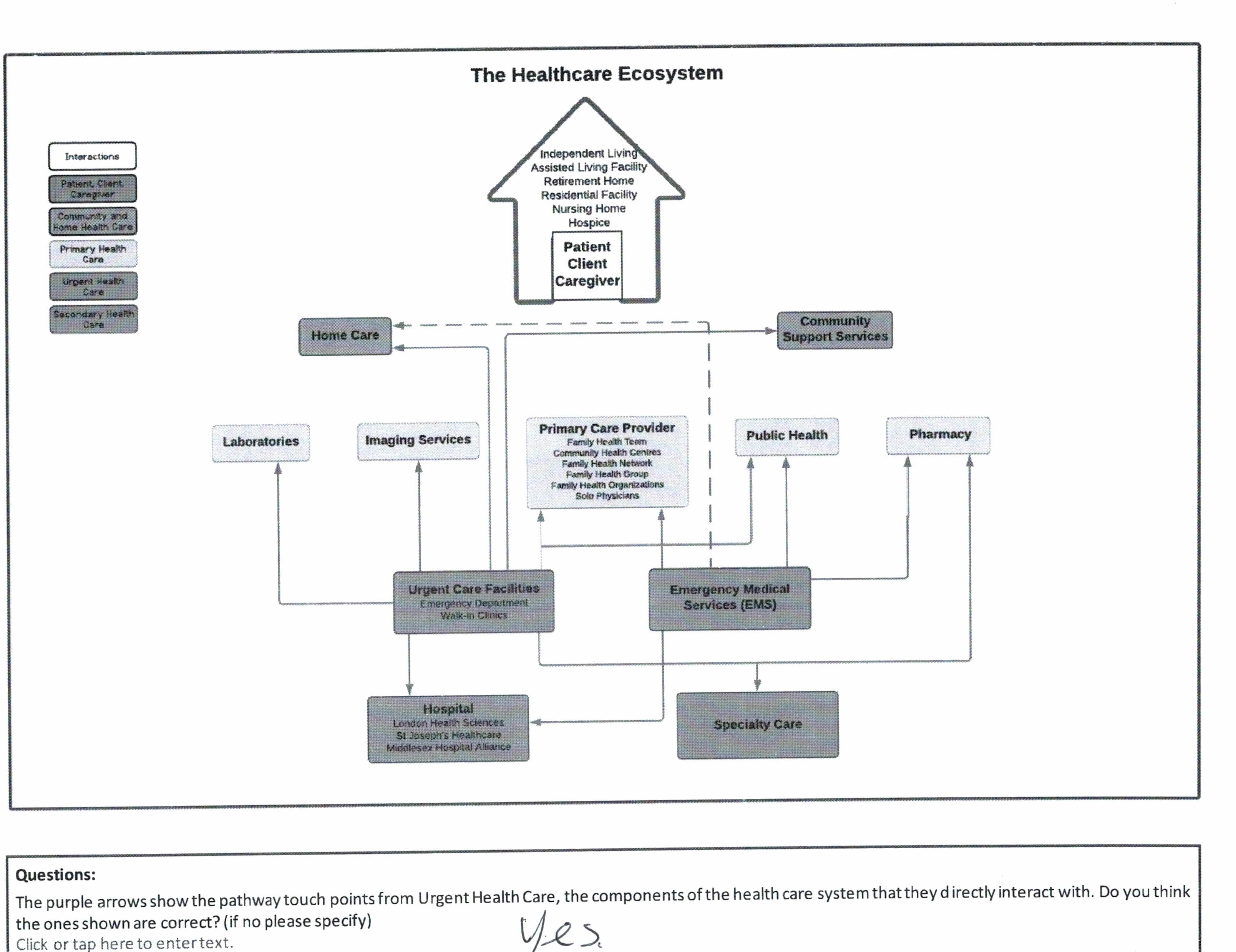


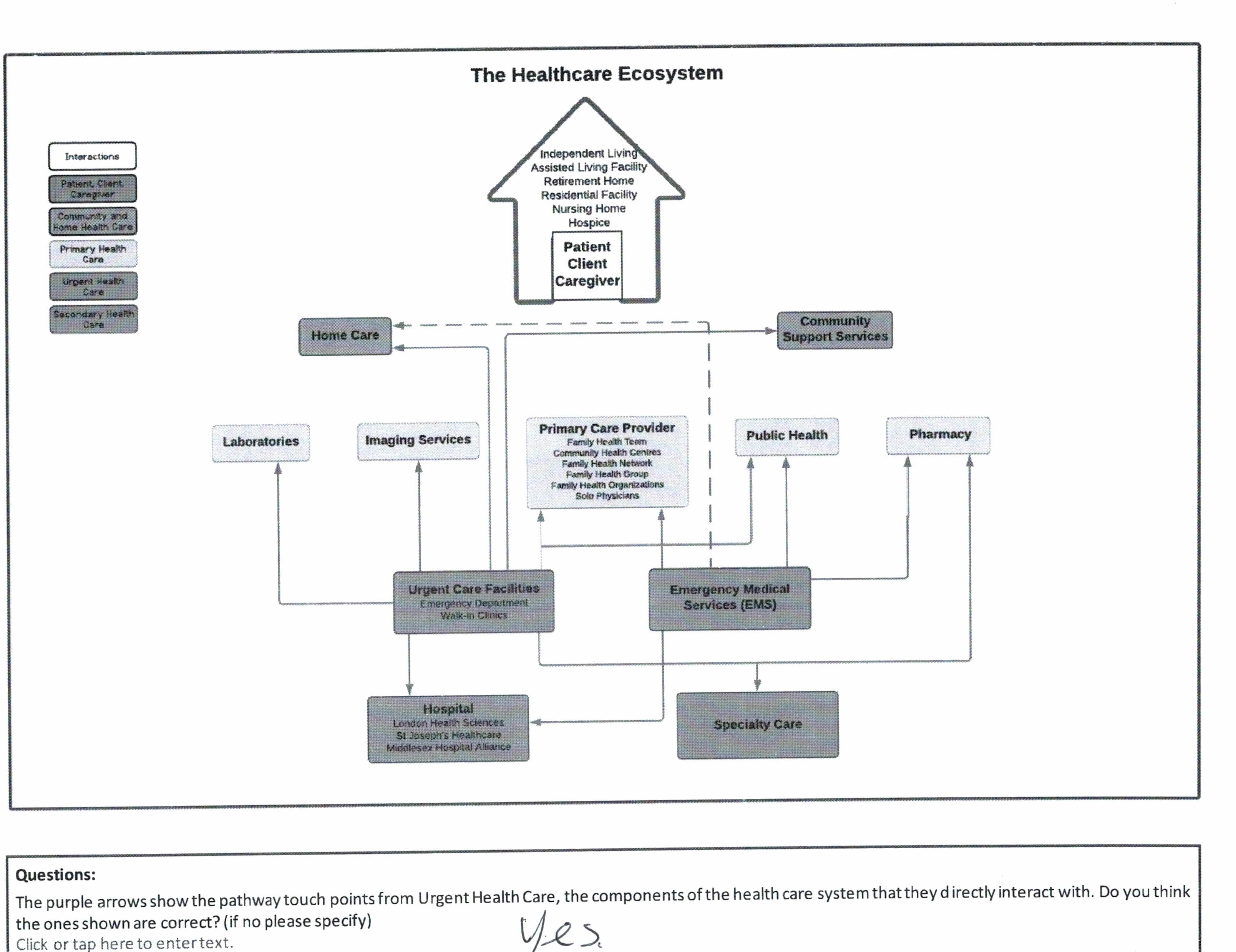

Supplement: Supplementary file 2 — Additional file 2. Step 4 questionnaires and feedback from 12 reviewers. [file 12913_2021_7168_MOESM2_ESM.docx]
